# Pushing the upper temperature limit of methanotrophy in continental hydrothermal ecosystems, active biological methane oxidation in hot springs of Yellowstone National Park

**DOI:** 10.3389/fmicb.2026.1736896

**Published:** 2026-03-23

**Authors:** Alta E. G. Howells, Michelle Santana, Ellen M. Cook, Brianna Orrill, Grayson Boyer, R. Vincent Debes, Kristopher M. Fecteau, Daniel R. Colman, Eric S. Boyd, Everett L. Shock

**Affiliations:** 1 Department of Geological Sciences, University of Colorado, Boulder, CO, United States; 2 Blue Marble Space Institute of Science, Seattle, WA, United States; 3 School of Life Sciences, Arizona State University, Tempe, AZ, United States; 4 Department of Geosciences, University of Cincinnati, Cincinnati, OH, United States; 5 School of Earth and Space Exploration, Arizona State University, Tempe, AZ, United States; 6 School of Molecular Sciences, Arizona State University, Tempe, AZ, United States; 7 Department of Microbiology and Cell Biology, Montana State University, Bozeman, MT, United States

**Keywords:** energy supply, hydrothermal ecosystems, methane oxidation rates, methanotrophy, Yellowstone National Park hot springs

## Abstract

Methane oxidation in terrestrial geothermal systems is an understudied process contributing to carbon cycling in extreme environments. We combined geochemical analyses, 16S rRNA gene amplicon sequencing, shotgun metagenome sequencing, and ^14^CH_4_ microcosm assays across 61 Yellowstone hot springs spanning pH 1.9–9.0 and temperatures of 28.6–92.2 °C to survey hydrothermal systems for methanotrophy. Bacterial aerobic methanotroph phylotypes were detected at multiple sites, including *Verrucomicrobia* (order S-BQ2-57) and *Alphaproteobacteria*, with the family *Methylocystaceae* having the highest relative abundance among bacterial methanotroph phylotypes. No known archaeal anaerobic methanotrophs were observed. Biological methane oxidation was widespread, occurring at 14 of 17 experimental sites under both ambient and air-amended conditions. Rates were highest at CH_4_-rich, NH_3_-poor sites dominated by bacterial methanotrophs, consistent with energy supply predictions integrating CH_4_/O_2_ and CH_4_/NH_3_ concentration ratios. Conversely, NH_3_-rich, energy-rich sites exhibited lower methane oxidation rates (MOR) and were dominated by archaeal ammonia oxidizers, primarily *Candidatus Nitrosocaldus*, suggesting chemical competitive inhibition of NH_3_ on methanotrophy. Remarkably, significant methane oxidation occurred at eight sites where no known methanotrophs were detected, including a site at 89.9 °C—well above the previously reported upper growth temperature limit for methanotrophs from continental geothermal and hydrothermal systems—pointing to uncharacterized thermophilic lineages. These results suggest that biological methane oxidation in Yellowstone hot springs is influenced by the interplay of substrate availability and energy supply. By linking energy supply calculations with microbial distributions, we identify both known methanotrophs (*Verrucomicrobia*, *Alphaproteobacteria*) and archaeal ammonia oxidizers as potential active contributors, while highlighting the potential for novel thermophilic lineages, thereby expanding the ecological and thermal boundaries of methane oxidation in extreme terrestrial ecosystems.

## 1 Introduction

Geologically derived methane is recognized as the fifth largest contributor to global methane emissions (Houghton et al., 2019), yet our understanding of its biological sinks in high-temperature continental systems remains limited. To date, only a few thermophilic methanotrophs (optimal growth >40 °C) have been cultivated from continental hydrothermal ecosystems (Houghton et al., 2019), leaving a significant gap in knowledge of the diversity, physiology, and ecological roles of methane-oxidizing microorganisms in these environments. Despite the scarcity of isolates, molecular surveys and rate measurements have demonstrated the presence of methanotrophs and methane oxidation activity in several continental hydrothermal and geothermal systems worldwide (Houghton et al., 2019). These findings suggest that methanotrophy may represent an important, yet underexplored, methane sink in extreme terrestrial habitats. Yellowstone National Park hosts one of the largest and most chemically diverse continental hydrothermal systems on Earth, yet no direct measurements of methane oxidation rates (MORs) have previously been reported from its hot springs. Establishing whether biological methane oxidation occurs in Yellowstone, and under what physicochemical conditions, is critical both for understanding the controls on methane cycling in terrestrial hydrothermal ecosystems and for constraining the upper temperature and pH limits of methanotrophy.

Methanotrophic activity has been measured in geothermal soils in New Zealand, Italy, and Canada with temperatures ranging from 22 to 83 °C with rates ranging from 2.4 to 99 μmol CH_4_·g^−1^·d^−1^ (Houghton et al., 2019). Methanotrophic activity has also been observed in hot springs in the Kuril Islands, Russia, with temperatures ranging from 40 to 99 °C (Kizilova et al., 2014), however, the activity measurement for the 99 °C system was determined at 75 °C. A recent study by Houghton et al. (2023) revealed that methane oxidation at elevated temperatures is more widespread in geothermal soils and hot springs in New Zealand than previously recognized. By incubating 57 soil microcosms drawn from 14 geothermal fields in Aotearoa-New Zealand, the authors demonstrated that both what they define as moderately thermophilic (>40 °C) and fully thermophilic (>60 °C) microbial methane consumption is common, with oxidation activity present in over half (54%) of the samples examined—with pH values ranging from 1.5–8.1 and temperatures 38–75 °C. Notably, oxidation rates varied widely (0.5–17.4 μmol CH_4_ g^−1^ d^−1^), and while some activity could be linked to known aerobic methanotrophs like *Methylacidiphilum* and *Methylothermus*, as well as putative anaerobic archaeal methanotrophs (Bathyarchaeota), a substantial portion of the activity occurred in the absence of known taxa or beyond known temperature niches. Enrichment and metatranscriptomic analyses further revealed unexpected community compositions and transcripts of genes associated with CH_4_ oxidation in *Methylococcaceae*, *Methylocystaceae*, and *Methylothermaceae*.

Multiple lines of evidence point to Yellowstone hot springs as viable habitats for methanotrophs. Thermodynamic modeling by Shock et al. (2010) and further supported by Robare (2025) demonstrated that aerobic methane oxidation is among the most favorable redox reactions available to chemolithotrophs in these systems, with energy yields of ~20–25 kcal per mole of electrons transferred. This makes it one of the strongest potential metabolic strategies in Yellowstone hot springs. Alternative pathways, such as methane oxidation coupled to nitrate or nitrite reduction, are also favorable but yield less energy (~15 kcal per mol e^–^), while coupling to sulfate reduction provides the lowest return (≤5 kcal per mol e^–^). Importantly, the thermodynamic window for sulfate-dependent methane oxidation narrows sharply with increasing pH, shifting from energy-yielding at acidic conditions to thermodynamically unfavorable above pH 6. Together, these results underscore that aerobic methane oxidation is feasible in Yellowstone hot springs, whereas alternative anaerobic pathways are constrained by both energy yield and geochemical context.

An additional line of evidence for methanotrophy in Yellowstone hot springs comes from shotgun metagenome sequencing. In a survey of the JGI IMG database (Chen et al., 2023), Houghton et al. (2019) identified seven metagenome-assembled genomes (MAGs) affiliated with known methanotrophic genera, including *Methylacidiphilum*, *Methylothermus*, *Methylococcus*, *Methylocystis*, and *Methylosinus*, across hot springs spanning 40–90 °C. Indeed, despite the presence of methanotroph MAGs in Yellowstone, their relatively sparse detection—against a backdrop of more than 100 hot spring metagenomes archived in IMG JGI (Chen et al., 2023)—suggests that additional geochemical or ecological constraints may strongly limit their abundance and activity. Thus, while metagenomics highlights the potential for methanotrophy, direct activity measurements are needed to establish the realized role of methanotrophs in Yellowstone hot springs.

Lastly, the strongest line of evidence for active methanotrophy in Yellowstone hot springs is the enrichment of a *Verrucomicrobia* methanotroph. Kim et al. (2022) applied a metagenomic sequencing approach to characterize a thermoacidophilic methanotroph enriched from a hot spring near Nymph Lake in Yellowstone National Park (conditions: 89.9 °C, pH 2.73). The enrichment was cultivated at 60 °C and pH 2, yielding a high-quality draft genome of *Candidatus Methylacidiphilum* sp. YNP IV (~2.47 Mbp, ~2,392 genes, ~41% GC content). Genomic analysis revealed genes involved in CH_4_ oxidation and assimilation, including those that encode particulate methane monooxygenase and CO_2_ fixation via the Calvin-Benson-Bassham cycle. Comparative genomics showed <95% average nucleotide identity with *M. fumariolicum* SolV, supporting its designation as a novel species and highlighting that acidic hot springs in Yellowstone can harbor bacterial methanotrophs.

In this study, we present the first survey of Yellowstone hot springs for biological methane oxidation activity. We implemented microcosm activity assays on sediments of hot springs in Yellowstone with ^14^CH_4_ to assess its conversion to ^14^CO_2_ and assimilation into biomass. These experiments provide evidence of biological methane oxidation under Yellowstone’s extreme hydrothermal conditions and expand the known environmental range where methane oxidation can occur. We conducted experiments across a wide spectrum of hydrothermal environments, encompassing sites where established methanotrophic lineages are expected to be active as well as sites where extreme physicochemical conditions challenge the known limits of biological methane oxidation. We quantified methane oxidation rates under hot spring conditions spanning pH values of 1.9–9.0 and temperatures of 38.5–89.8 °C. This temperature range not only exceeds the upper cultivation limit of methanotrophs isolated from continental hydrothermal systems by 20.2 °C but also surpasses previously measured activity in such systems by 17.2 °C. Beyond activity measurements, we coupled these assays with detailed characterization of substrate concentrations and energy supplies, providing new insights into the metabolic feasibility of methanotrophy under extreme conditions. By integrating energy supply constraints with microbial distribution patterns and activity, we shed light on the environmental conditions that may enable biological methane oxidation in Yellowstone hot springs, as well as the chemical and biological interactions that shape the ecological boundaries of methanotroph activity.

## 2 Methods

### 2.1 Site description

Sampling for geochemistry and microbiome analysis and execution of microcosm experiments to characterize rates of methanotrophy in Yellowstone hot spring systems was carried out in the summer of 2014, July 24th to August 5th, and in one location (Geyser Creek) in July 2015 (23rd and 24th). The sampling date for each study site is reflected in the “Site ID” of Table 1 (date represented by year-month-day annotation). Also shown in Table 1 are the sampling locations, pH, temperature, and biological analyses done for each site. We note in Table 1 the sampling sites whose geochemistry and 16S rRNA gene phylotype composition were previously reported by Weeks et al. (2023).

**Table 1:** Overview of sampling sites with sampling location, in-field measurements of sampling site fluid temperature, pH, and the type of biological analysis conducted for each site.

Site ID	Area	Temp. (°C)	pH	Biological analysis
140724SC*	Geyser Creek	50.0	8.0	16S + MOR
140724SD*	Geyser Creek	54.8	7.7	16S
140724SE*	Geyser Creek	70.2	7.1	16S
140725SI*	Sentinel Meadows	59.2	9.0	16S
140725SJ*	Sentinel Meadows	64.6	9.0	16S
140725SK*	Sentinel Meadows	75.4	8.9	16S
140725SL*	Sentinel Meadows	67.9	9.0	16S
140726SO*	GOPA	50.9	6.1	16S
140726SP*	GOPA	58.8	5.8	16S
140726SQ*	GOPA	64.1	5.2	16S
140726SR*	GOPA	43.7	6.5	16S
140727SU*	Crater Hills	46.3	3.4	16S
140727SV*	Crater Hills	50.3	1.9	16S
140727SW*	Crater Hills	53.0	2.0	16S
140727SX*	Crater Hills	58.8	1.9	16S
140727SY*	Crater Hills	50.8	1.9	16S
140727SZ*	Crater Hills	57.5	1.9	16S
140729SD*	Crater Hills	28.6	3.6	16S
140729SE*	Crater Hills	28.8	3.7	16S
140730SI*	Sentinel Meadows	68.0	8.3	16S + MOR
140730SJ*	Sentinel Meadows	69.3	8.2	16S
140730SK*	Sentinel Meadows	73.1	8.1	16S + MOR
140730SL*	Sentinel Meadows	92.2	7.6	16S
140730SN*	Sentinel Meadows	83.5	7.9	16S + MOR
140731SP*	Sylvan Spring Area	44.4	2.5	16S
140731SQ*	Sylvan Spring Area	47.1	2.5	16S
140731SR*	Sylvan Spring Area	49.7	2.5	16S
140802SB*	Rabbit Creek-North	73.0	9.0	16S
140802SC*	Rabbit Creek-North	78.2	9.0	16S + MOR
140803SG*	Amphitheater Springs	50.9	2.3	16S
140803SH*	Amphitheater Springs	52.8	2.3	16S
140803SI*	Amphitheater Springs	74.0	2.4	16S
140803SJ*	Amphitheater Springs	53.5	2.3	16S
140803SO*	Amphitheater Springs	44.1	2.4	16S
140803SP*	Amphitheater Springs	62.3	2.4	16S
140804SS*	Norris Geyser Basin	33.2	5.6	16S
140804st*	Norris Geyser Basin	53.9	5.7	16S
140804SU*	Norris Geyser Basin	66.8	5.5	16S
140804SV*	Norris Geyser Basin	77.5	3.9	16S
140805SA*	Rabbit Creek-North	72.8	8.8	16S
140805SB*	Rabbit Creek-North	76.9	8.8	16S
140805SC*	Rabbit Creek-North	66.8	8.7	16S
140805SD*	Rabbit Creek-North	70.8	8.8	16S
140805SE*	Rabbit Creek-North	77.6	8.6	16S
140805SZ*	Rabbit Creek-North	69.5	8.9	16S
150723MC	Geyser Creek	38.9	6.7	16S
150724MA	Geyser Creek	34.7	3.1	16S
150724MB	Geyser Creek	59.4	7.9	16S
150724MC	Geyser Creek	39.4	7.0	16S
140725TI	GOPA	74.0	5.4	16S + MOR
140725TK	GOPA	66.8	6.0	16S + MOR
140726SN	GOPA	65.9	6.5	16S + MOR
140727TT	Washburn	89.5	6.0	16S + MOR
140727TU	Washburn	57.6	6.4	16S + MOR
140729SB	Crater Hills	78.1	2.2	16S + MOR
140731SS	Sylvan Spring Area	75.7	1.9	16S + MOR
140731ST	Sylvan Spring Area	38.5	3.5	16S + MOR
140803TA	Calcite	89.9	7.3	16S + MGS + MOR
140804SR	Norris Geyser Basin	85.8	7.3	16S + MOR
140805TJ	Rabbit Creek-South	52.9	4.0	16S + MOR
140805TL	Rabbit Creek-South	45.7	4.3	16S + MOR

*Geochemical data and 16S rRNA gene sequencing reported by Weeks et al. (2023).

16S, rRNA gene amplicon sequencing; MGS, Shotgun metagenome sequencing; MOR, Microcosms for methane oxidation rate; GOPA, Greater Obsidian Pool Area.

**Figure 1:**
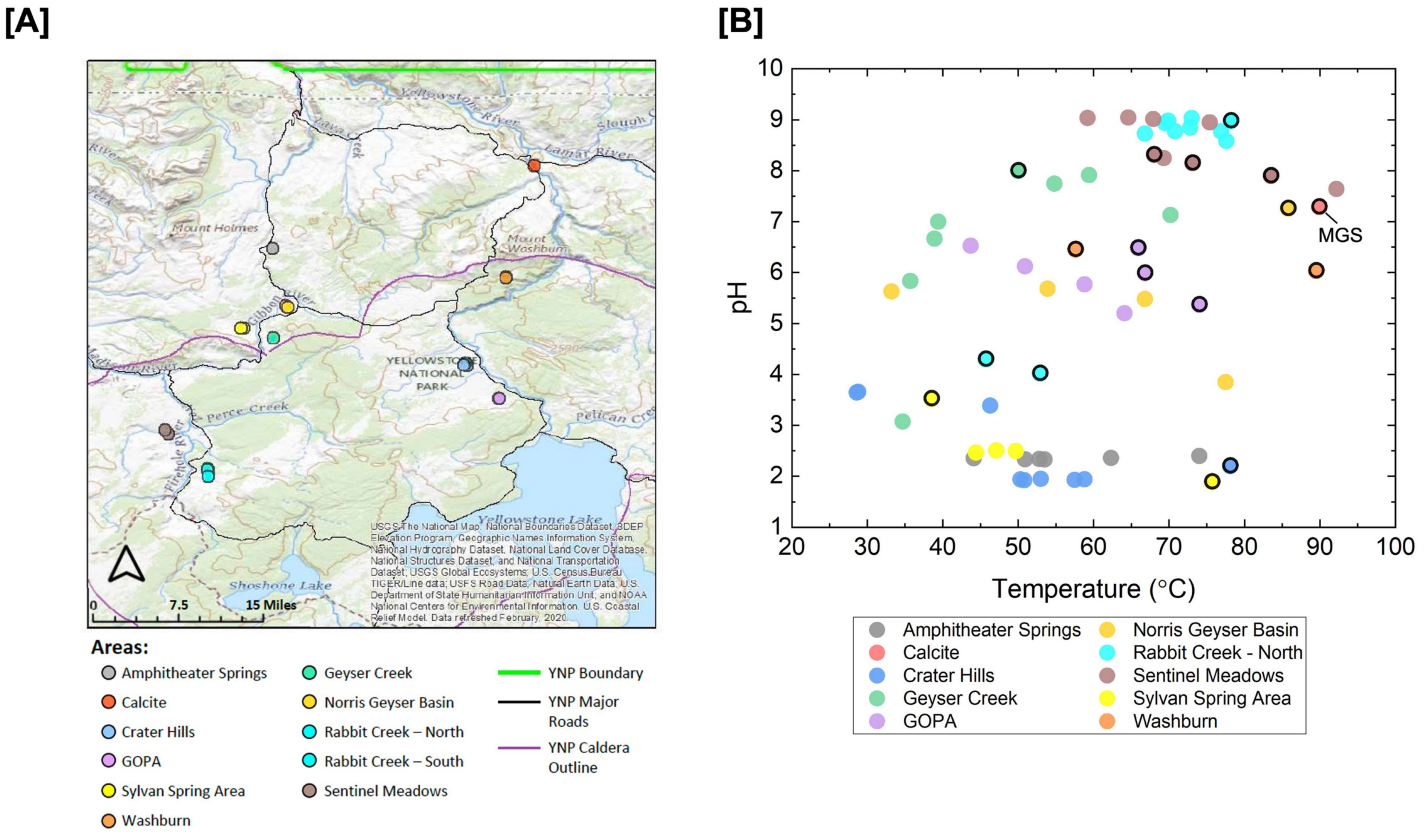
Topographical map of Yellowstone National Park and hot spring sampling site pH and temperature. The base map is from the USGS. This map was created using ArcGIS^®^ software by Esri. ArcGIS^®^ and ArcMap™ are the intellectual property of Esri and are used herein under license. Copyright © Esri. All rights reserved. Geospatial data for YNP boundary, roads and caldera sourced from the US National Park Service and Yellowstone National Park. The locations of the study sites are indicated by the different colored points on **(A)**. Field pH and temperature of the springs where 16S rRNA gene sequencing was conducted are indicated by circles, and experimental sites (outlined circles) are shown in **(B)**. 16S rRNA gene sequencing was also conducted on sediment samples from experimental sites. The locations shown in **(A)** are indicated in **(B)** by the color of the symbols. “MGS” indicates the site in **(B)** where shotgun metagenome sequencing was also conducted on DNA extracts from sediment samples. “GOPA” is Greater Obsidican Pool Area.

We sampled across Yellowstone in the locations Amphitheater Springs, Calcite, Crater Hills, Greater Obsidian Pool Area (GOPA), Sylvan Spring Area, Washburn, Geyser Creek, Norris Geyser Basin, Rabbit Creek-North, Rabbit Creek-South, and Sentinel Meadows as shown on the geological map depicted in Figure 1A. Overall, 61 sites were sampled for geochemical analysis and a 16S rRNA gene amplicon sequencing survey under research permit YELL-SCI-5434 from the Yellowstone Center for Resources. The pH and temperature of the sampling sites (pH 1.9–9.0, 26.6–92.2 °C) are depicted in Figure 1B. At 17 of 61 16S rRNA gene sequencing sites outlined in black in Figure 1B, microcosm experiments were conducted to measure rates of biological methane oxidation. At one site, indicated by “MGS” in Figure 1B, a sample was collected for shotgun metagenome sequencing.

Fluid samples for geochemical analyses and samples collected for biological analyses (16S rRNA gene sequencing, metagenomic sequencing) and microcosm experiments were collected concurrently on the same day. Sampling sites were selected based on prior geochemical characterizations of Yellowstone hot spring fluids reported in Shock et al. (2010), with an emphasis on methane concentrations, electron acceptor availability, energy constraints, and other chemical parameters relevant to methanotrophic activity.

### 2.2 Field measurements and geochemical sample collection

Temperature, pH, and conductivity were measured on site following previously published protocols (Boyer et al., 2020; Weeks et al., 2023). Temperature and conductivity were recorded using a YSI-30 handheld meter (YSI, Yellow Springs, OH, United States). pH was measured with a WTW3110 meter equipped with SenTix 41 temperature-compensated probes (Xylem Analytics, Weilheim, Germany), calibrated daily at ambient temperature using standard pH buffers. Dissolved oxygen was quantified optically with a PreSens Fibox 4 meter and DP-PSt3-L2.5-St10-YOP-HT sensor, calibrated to 100 °C, as described in St Clair et al. (2019). Total dissolved sulfide was determined immediately after sampling on unfiltered water using the methylene blue method (Hach method 8131) with a Hach DR1900 spectrophotometer. Dissolved ferrous iron was also determined after filtering using the 1,10 phenanthroline method and a Hach spectrophotometer.

Filtered water samples (0.2 μm Supor membrane filters; Pall Corporation, Port Washington, NY, United States) were collected and stored according to Fecteau et al. (2022). Anion samples were collected and stored in High Density Polyethylene (HDPE) bottles (30 mL Nalgene) that were triple rinsed, soaked overnight, triple rinsed again, and went into the field filled with 18.2 MΩ-cm ultrapure deionized (DI) water. Cation samples were collected and stored in HDPE bottles (30 mL Nalgene) prepared with the same DI water rinse, soak, rinse protocol described for anion samples, with the final step being replaced with drying under laminar flow and a 6 M methanesulfonic acid spike to achieve a final concentration of ~20 mM after filling with spring water. Both anion and cation sample bottles were frozen at −20 °C until analysis. Dissolved inorganic carbon (DIC) samples were collected in nitric acid-washed 40 mL amber glass vials with no headspace, sealed with nitric acid-washed black butyl rubber septa, and stored at 5 °C until analysis. Dissolved gas samples for CH_4_ analysis were collected in triplicate using the same methods described in Canovas et al. (2017). For field samples, 50 mL of water was equilibrated with 10 mL of carrier gas (100% N_2_). Samples were stored in 100 mL Cali-5-Bond bags made by Calibrated Instruments.

### 2.3 Chemical laboratory analyses

Anions (F^−^, Cl^−^, SO_4_^2−^, Br^−^, NO_3_^−^, NO_2_^−^) and cations (Li^+^, Na^+^, K^+^, Mg^2+^, Ca^2+^, NH_4_^+^) were analyzed using separate Dionex DX-600 ion chromatography systems (4 mm) with suppressed conductivity detection as previously described (Weeks et al., 2023). Dissolved inorganic carbon (DIC) concentrations were measured using an OI Wet Oxidation TOC analyzer coupled to a Thermo Delta Plus Advantage mass spectrometer (Havig et al., 2011). CO_2_ was produced via phosphoric acid addition and quantified using the 44 m/z molecular ion peak against sodium bicarbonate (DIC). Three loop sizes—1 mL (10–200 mg C L^−1^), 5 mL (2–50 mg C L^−1^), and 25 mL (0.25–8 mg C L^−1^)—were used to accommodate varying DIC concentrations across samples.

Methane (CH_4_) was measured using gas chromatography (GC) equipped with a flame ionization detector (FID; Peak Laboratories), operated with research grade nitrogen (99.99995%) carrier gas and air and ultra-high-purity hydrogen for the detector flame. Standard curves were made by injections of 5 ppm CH_4_ standards over a range of injection volumes. From the 10 mL gas sample, 250 μL subsamples were injected into the GC–FID for CH_4_ determination. The reproducibility of replicate sample injections was within ±5%. Supplementary Table 1 summarizes field and laboratory measurements.

### 2.4 Energy calculations

Energy supplies in units of calories per kg of fluid for the reactions summarized in Table 2 were calculated using the methods described by Howells et al. (2025). For this study, estimations of chemical activities and calculations of energy supplies were done using an early version of the AqEquil Python package (Boyer et al., 2024), a program wrapper for EQ3/6 (Wolery, 2013) which is now hosted on the Water-Organic-Rock-Microbe (WORM) Portal^1^. For chemical speciation, Cl^−^ was used for charge balance, redox reactions were suppressed, and the fugacity of oxygen of each sample was set by the concentration of dissolved O_2_.

**Table 2:** A list of potential methane oxidizing and taxa and summary redox reactions for their metabolisms.

Microbial methane oxidizing group	Redox reaction
Bacterial aerobic methanotrophs (*Alphaproteobacteria, Gammaproteobacteria, Verrucomicrobia*)	CH_4_ + 2O_2_ → CO_2_ + 2H_2_O
Aerobic ammonia oxidizers (archaeal and bacterial)	CH_4_ + 2O_2_ → CO_2_ + 2H_2_O
NC10 bacterial anaerobic methanotrophs	3CH_4_ + 8NO_2_^−^ + 8H^+^ → 3CO_2_ + 4N_2_ + 10H_2_O
ANME (anaerobic archaeal methanotrophs)	CH_4_ + 4NO_3_^−^ → CO_2_ + 4NO_2_^−^ + 2H_2_O
ANME (anaerobic archaeal methanotrophs)	CH_4_ + SO_4_^−2^ + 2H^+^ → CO_2_ + H_2_S + 2H_2_O
Aerobic alkane oxidizers	CH_4_ + 2O_2_ → CO_2_ + 2H_2_O
Unknown methanotrophs	Many possible reactions

For reactions involving NH_3_ or NH_4_^+^, chemical activities derived from total ammonia (∑NH_4_^+^, NH_3_) were used for calculations of energy supply. When comparing energy supplies with NH_3_ or NH_4_^+^ concentration, we use chemical activities for concentration in molality (activity ≈ molality) assuming that the difference between chemical activity and molality is negligible given that the ionic strength of the sampling sites varies between 0.002 and 0.048 (average 0.019 ± 0.009). Supplementary Table 2 summarizes the chemical activities of reactants and products and energy supplies for reactions listed in Table 2.

### 2.5 DNA sample collection and extraction

For a broad 16S rRNA gene amplicon survey, DNA samples were collected alongside geochemical measurements at 49 sites, as previously reported by Weeks et al. (2023). An additional 17 sites were sampled in parallel with material collected for microcosm experiments; five of these sites overlapped with the broad survey (140724SC, 140730SI, 140730SK, 140730SN, and 140802SC) and therefore represent replicate analyses. In total, 61 unique sites were characterized for both 16S rRNA gene sequencing and geochemistry, yielding 66 sequencing datasets, including five replicate analyses. Immediately following sampling for geochemical analyses, samples for DNA analyses were collected. Sediment samples were collected either with a sterile stainless-steel spatula or a sterile Teflon scoop to fill a sterile 50 mL specimen cup. Once in the specimen cup, the sediment sample was homogenized. Three 1.8 mL sterile, DNAse/RNAse-free cryo vials were filled with sediment for DNA extraction and frozen on dry ice in the field. DNA sediment samples were stored on dry ice while transferring samples to the lab. Upon arrival to the lab, sediment samples were stored at −80 °C until DNA extraction.

DNA extraction was carried out using the ZymoBIOMICS DNA Miniprep Kit (Catalog # D4300), binding capacity 25 μg. The purity of the DNA sample was evaluated spectrophotometrically using a NanoDrop. DNA was quantified using a Qubit fluorometric assay kit from Invitrogen (Cat. # Q32850).

### 2.6 16S rRNA gene amplicon sequencing and analysis

Amplicon sequencing of 16S rRNA genes was performed at the Arizona State University Biodesign Institute using the Illumina MiSeq v3 platform (2 × 300 bp). Libraries were prepared with a two-step PCR and dual-indexing approach (Kozich et al., 2013). For 16S rRNA genes, the first PCR used the Earth Microbiome Project primers, 505F and 806R (Caporaso et al., 2011; Apprill et al., 2015; Parada et al., 2016). First-step PCR conditions for 16S rRNA gene amplicons were: 94 °C for 3 min, 30 cycles of 94 °C for 30 s, 48 °C for 30 s, 72 °C for 50 s, and a final 72 °C for 10 min. The second PCR used 95 °C for 3 min, 10 cycles of 95 °C for 30 s, 50 °C for 30 s, 72 °C for 50 s, and a final 72 °C for 10 min. Each 30 μL reaction contained 15 μL EconoTaq 2 × Master Mix, 2.5 μL of each primer (2 μM), 2.5 μL template DNA, and 7.5 μL nuclease-free water. PCR products were purified using Ampure magnetic beads (Beckman Biomek NXp) and quantified with Qubit dsDNA dye and a Biotek HT1 Plate Reader. Volumes containing equal masses (25 ng) of uniquely barcoded amplicons were pooled for sequencing. All raw sequence data are publicly available in the NCBI Sequence Read Archive under BioProject accession PRJNA938133 (previously reported on by Weeks et al. (2023)) and BioProject PRJNA1437826 (this study).

Bioinformatic processing was performed in QIIME2 (v2019.7; RRID: SCR_021258; Bolyen et al., 2019). Paired-end reads were demultiplexed at the Arizona State University Genomics Facility and returned in Casava 1.8 format as compressed FASTQC files (.fastqc.gz) with accompanying quality reports. Amplicon sequence variants (ASVs) were generated using the DADA2 pipeline (Callahan et al., 2016), which included quality filtering, trimming of low-quality bases, Illumina error correction, read merging, and chimera removal. Taxonomic classification was carried out with a naïve Bayes classifier trained on the SILVA 132 database at 99% operational taxonomic unit (OTU) identity for 16S rRNA genes (Quast et al., 2012), using the q2-feature-classifier plugin and the sklearn method (Pedregosa et al., 2011; Bokulich et al., 2018). To reduce spurious detections, singleton 16S ASVs occurring fewer than 10 times across all samples were removed. Additionally, ASVs classified only to the domain level and those matching known human-associated microbiota were excluded as well as ASVs associated with chloroplasts. Post-filtering, sequencing depth was normalized by rarefaction to ensure even sampling across sites (3,000 reads per sample). Alpha rarefaction curves were generated to confirm adequate sampling coverage. The resulting rarefied datasets were used to build ASV frequency tables for diversity analyses. ASVs classified as previously characterized aerobic bacterial methanotrophs and aerobic archaeal ammonia oxidizers and their counts across all hot spring sites can be found in Supplementary Tables 3, 4, respectively. The relative abundance of ASVs grouped at the species level across all sites can be found in Supplementary Table 5. The metabolic potential associated with aerobic methanotroph and aerobic ammonia oxidizer phylotypes was inferred by ensuring that all previously characterized members of their taxonomic level of classification in the National Center for Biotechnology Information Taxonomy database^2^ have been shown to carry out that metabolism.

### 2.7 DNA extraction and shotgun metagenome sequencing of a high temperature, hydrocarbon-rich site

DNA was extracted from 4.2 grams of sediment from site 140803TA (“MGS” in Figure 1B). DNA extraction was carried out using the ZymoBIOMICS DNA Microprep Kit (Catalog # D4301). From a total of 8 replicate DNA extractions, 170 ng of DNA was pooled. 60 ng was sent to the Marine Biological Laboratory at Woods Hole Oceanographic Institute for shotgun metagenome sequencing. The Illumina 150 × 2 with standard protocol was used. Raw sequencing data was uploaded to the National Center for Biotechnology Information Sequence Read Archive under the Bioproject, https://www.ncbi.nlm.nih.gov/bioproject/PRJNA1427733.

Raw DNA sequencing data files were uploaded into KBase^3^ for processing. Read files were quality-trimmed with Trimmomatic v0.36 (Bolger et al., 2014) using a sliding window size of 4 and a minimum quality score of 25. Read quality was then evaluated with FastQC v0.12.1^4^, and high-quality reads were assembled using metaSPAdes v3.15.3 (Nurk et al., 2017) with a minimum contig length of 1,000 bp and a minimum read length of 300 bp. This assembly produced 8,342 contigs, which were annotated using RASTtk v1.073 (Brettin et al., 2015) with default KBase parameters.

Contigs were binned using CONCOCT v1.1 (Alneberg et al., 2014) (minimum length: 1,000 bp; maximum length: 2,500 bp) and MetaBAT2 v1.7 (Kang et al., 2015) (minimum length: 1,500 bp; maximum length: 2,500 bp). CONCOCT binned 3,518 contigs into 30 bins, while MetaBAT2 binned 2,245 contigs into 22 bins. Bin sets from both approaches were refined using DAS Tool v1.1.2 (Sieber et al., 2018) with default parameters, producing 17 bins from 2,173 contigs.

Bin quality was assessed with CheckM v1.0.18 (Parks et al., 2015), confirming >65% completeness and <3% contamination for all bins. Bins were extracted as assemblies with Binned Contigs v1.0.2 in Kbase and annotated using DRAM v0.1.2 (Shaffer et al., 2020) (minimum contig length: 1,000 bp; metagenome mode; default parameters). Taxonomic classification was performed with GTDB-Tk v1.7.0 (Chaumeil et al., 2022) to the order and, when possible, family/genus levels. Check M results and GTDB-Tk annotations of bins can be found in Supplementary Table 6.

Fasta files from DRAM annotations were manually screened for monooxygenase enzymes. All associated amino acid sequences were queried against the NCBI BLASTp (Camacho et al., 2009) database, and results were compiled into an information matrix including: bin number, DRAM enzyme ID, NCBI enzyme ID, GTDB-Tk taxonomic classification, NCBI taxonomic classification, percent identity, *e*-value, and query coverage. This matrix was cross-referenced with taxa reported to degrade hydrocarbons (Chamy and Rosenkranz, 2013) to identify hydrocarbon-degrading microorganisms in the dataset.

### 2.8 Survey of Yellowstone hot spring MAGs for monooxygenases

Using MAGs and methods described by Colman et al. (2024) we carried out a survey for the presence of genes that encode potential monooxygenase proteins. Putative metabolic functions were identified using the METABOLIC (Zhou et al., 2022) pipeline, which employs a suite of hidden Markov model profiles to annotate genes involved in key biogeochemical pathways. Annotated functions identified via METABOLIC were used as an initial framework to assess metabolic potential across MAGs from Colman et al. (2024). This approach specifically allowed us to identify genes encoding putative particulate methane monooxygenase (*pmoA*), ammonia monooxygenase (*amoA*), soluble methane monooxygenase (*mmoB*), and divergent methyl co-enzyme M reductase (*mcrA*).

To further evaluate the presence of alkane and nitronate monooxygenase homologs, representative protein sequences in the database described by Wang et al. (2023) were used as queries in BLASTp searches against all predicted protein-coding genes within the MAGs. Homologs were considered putative positive hits when they exhibited >60% query coverage and >30% amino acid identity to the reference sequences. These criteria ensured a balance between stringency and inclusivity in detecting functional homologs potentially involved in hydrocarbon and nitroalkane oxidation processes. A summary of the presence/absence and relative abundance of genes encoding putative monooxygenases across Yellowstone hot spring systems studied by Colman et al. (2024) can be found in Supplementary Table 7.

### 2.9 Production of ^14^CH_4_

The same methods described by Harder (1997) and Zehnder et al. (1979) were used for the biological production of ^14^CH_4_, however a different methanogen, *Methanospirillum hungatei* JF1, was used. The methanogen strain, *Methanospirillum hungatei* JF1, was cultivated using the growth medium and conditions described by McInerney et al. (1979) for a seed culture in the lab of Hinsby Cadillo-Quiroz at Arizona State University. For the production of ^14^CH_4_, the organism was switched to a growth media buffered by 20 mM HEPES, pH 7, instead of carbonate buffer to ensure that the organism would use the supplied H_2_ and ^14^C-NaHCO_3_ as methanogenesis substrate. Five cultures were grown for generating ^14^CH_4_ for use in downstream experiments. ^14^CH_4_ was harvested from the cultures using methods described by Harder (1997). Briefly, after cultures grew for 5 days at 30 °C, 10 mL of 10 M NaOH reduced with dithionite was added to each vial and incubated for 20 min. After incubation the headspace of the vials was displaced with saturated NaCl solution with dithionite. The headspace gas was collected in a 60 mL plastic syringe with a two-way stopcock and injected into pre-vacuumed 56 mL vials containing 6 g hopcalite to convert residual ^14^CO to ^14^CO_2_. The ^14^CO_2_ produced was trapped by adding 5 mL 10 M NaOH and incubated for 20 min. The headspace of the vials containing hopcalite were then displaced with saturated NaCl solution containing 1 M NaOH. Headspace gas of vials containing the produced ^14^CH_4_ were collected in a 60 mL plastic syringe and stored in 100 mL volume Cali-5-Bond bags (Calibrated Instruments). All solutions used for gas transfer were sparged with 100% N_2_ for 30 min to remove atmospheric CO_2_ prior to use. The activity of the ^14^CH_4_ that was produced was determined using scintillation counting (Tri-Carb 2900TR Liquid Scintillation Analyzer, Perkin Elmer) according to the methods described by Zehnder et al. (1979). The average disintegrations per minute (DPM) of the five bags of ^14^CH_4_ generated from five cultures was 1,800,000 ± 400,000. To quantify the production of CH_4_, triplicate cultures with additions of the same amount of NaHCO_3_, but unlabeled, were grown under the same conditions for ^14^CH_4_ production. Unlabeled cultures were started with the same seed culture as labeled cultures and were grown at the same time as the labeled cultures. CH_4_ was harvested from the cultures and stored in the same way as the ^14^CH_4_. The gas composition of the bags was evaluated using a thermal conductivity detector. The average partial pressure of CH_4_ was 1.6% ± 0.3% by volume with N_2_ as the balance. Remnant CO_2_ was less than 0.0005%.

### 2.10 Microcosms with ^14^CH_4_

Microcosm experiments were conducted in 20 mL serum vials sealed with Chemglass blue 20 mm butyl rubber stoppers (CLS-4209-14), 10 mL of hot spring fluid, and less than 1 gram of sediment per experiment. In the field, two experimental conditions were conducted in triplicate, one with the headspace gas composition in equilibrium with what was dissolved in the hot spring waters, labeled “ambient,” and one that was “air-amended.” For experiments with ambient gas composition, less than 1 g of the homogenized sediment sample was added to each vial. The vials were sealed and then purged for 10 min with 100% N_2_ gas to remove atmospheric gasses. Hot spring fluid was collected from the hot spring sampling site using a peristaltic pump (rinsed thoroughly between experiments). The peristaltic pump line fed into a 60 mL syringe. From there, maintaining gas tight conditions, 10 mL of hot spring fluid was added to the sealed vials. Air-amended experiments were conducted in the same manner as ambient experiments except hot spring fluid was added without purging with N_2_ in advance. Therefore, atmospheric gasses remained in the system. Fluids for microcosm experiments were collected immediately after dissolved gas sampling (Section 2.2) to ensure consistency with gas composition analyses and to account for potential temporal variability in dissolved gas concentrations. Killed controls were prepared in the same manner as air amended experiments save for the addition of 100 μL of 6 M NaOH. After addition of fluid, 1 mL of the ^14^CH_4_ gas produced as described above was added to each vial. Vials were wrapped in aluminum foil to prevent potential photooxidation of methane and incubated in the hot spring for 2–4 h. After incubation, vials were immediately put on dry ice and stored at −20 °C until processing. This work was conducted under research permit YELL-2014-SCI-5544.

Methods modified from those of Harder (1997) were used for processing experimental vials. Briefly, any ^14^CO_2_ generated during the experiment was trapped by adding 1 mL of 4 M NaOH to the serum vial and incubating overnight. From there, remaining ^14^CH_4_ was removed by sparging with N_2_ gas for 1 h. After sparging, still-sealed vials were acidified with 1 mL of 12 M HCl and incubated for 1 h with shaking every 10 min. After acidification, 4 mL of vial headspace, now containing the ^14^CO_2_, was added to 10 mL sealed vials containing 1 mL Carbo-Sorb^®^E and incubated for 2 h. After incubation, the Carbo-Sorb^®^E containing the sample was added to scintillation vials with 10 mL CytoScint ES liquid scintillation cocktail and analyzed on a scintillation counter (Tri-Carb 2900TR Liquid Scintillation Analyzer, Perkin Elmer).

To analyze assimilation of ^14^CH_4_ into biomass, methods described by Urschel et al. (2015) were used. Briefly, the acidified vials were opened and sediments from the experiment were collected on 0.22 mm-pore-size polycarbonate filters and washed with 5 mL sterile DI water. The filtered sediments were dried overnight at 80 °C. Before and after filtering, the filter was weighed to obtain the grams of dry weight sediment (gdws) value. The filter was then placed in a scintillation vial, 10 mL CytoScint ES liquid scintillation cocktail was added, and the sample was analyzed by scintillation counting (Tri-Carb 2900TR Liquid Scintillation Analyzer, Perkin Elmer).

The MOR was calculated following Bastviken et al. (2002) with the formula:


$$\mathrm{MOR}=\frac{\left({\mathrm{DPM}}_{\exp}/{\mathrm{DPM}}_{\text{added}})[{\mathrm{CH}}_{4}\right]}{t}$$

where DPM_added_ is the radioactivity of ^14^CH_4_ added to each experiment, DPM_exp_ is the average of the measured radioactivity from ^14^CO_2_ or ^14^C assimilated into biomass during the experiment of the biological replicates minus the average DPM of killed controls, [CH_4_] is the concentration of CH_4_ in the hot spring fluid and *t* is the incubation time. MOR is calculated for CH_4_ oxidation to CO_2_ and assimilation into biomass separately and then added to get a total MOR.

## 3 Results and discussion

### 3.1 Geochemical and molecular investigation of methane oxidation in YNP hot springs

To examine biological methane oxidation potential in Yellowstone hot springs, putative methane-oxidizing taxa were identified via 16S rRNA gene amplicon sequencing and phylogenetic inference. Table 3 lists these taxa along with the total frequency of ASVs detected across sites. No known anaerobic methanotrophs were detected. Among aerobic methanotrophs, *Alphaproteobacteria* and *Verrucomicrobia* were represented, with *Alphaproteobacteria* ASVs classified as *Methylocystaceae* having the highest frequency across all sites (588) with a maximum relative abundance (16.4%) at site 150724MA (see Supplementary Table 5). The most frequently detected *Verrucomicrobia* ASVs (10, see Table 3) were classified within the order S-BQ2-57, with the highest relative abundance (0.2%) at site 140724SC. Table 3 also lists the ammonia oxidizers detected. No known bacterial ammonia oxidizers were present, but archaeal ammonia oxidizers were detected, with ASVs classified as the genus “*Candidatus* Nitrosocaldus” having the highest frequency (596, see Table 3) and highest relative abundance (5.2%) at site 140724SE. Overall, bacterial aerobic methanotrophs were detected in only 12 of the 61 sequencing sites, whereas archaeal aerobic ammonia oxidizers were detected in 25 sites (see Supplementary Table 5).

**Table 3:** Frequency of putative methane oxidizing taxa ASVs detected through 16S rRNA gene amplicon sequencing in this study.

Putative methane oxidizing taxa	Frequency of ASVs
Methane oxidizing bacteria	
Class, *Alphaproteobacteria*:	
Family, *Methylocystaceae*	588
Class, *Verrucomicrobia*:	
Order, S-BQ2-57	10
Order, Methylacidiphilales	4
Species, “*Candidatu*s Methylacidiphilum infernorum”	3
Family, LD19	1
Genus, “*Candidatus* Methylacidiphilum”	1
Ammonia oxidizing archaea	
Genus, “*Candidatus* Nitrosocaldus”	596
Species, “*Candidatu*s Nitrososphaera SCA1145”	23
Species, “*Candidatus* Nitrososphaera gargensis”	3
Phylum, Thaumarchaeota	2
Family, *Cenarchaeaceae*	2
Order, D-F10	1

**Figure 2:**
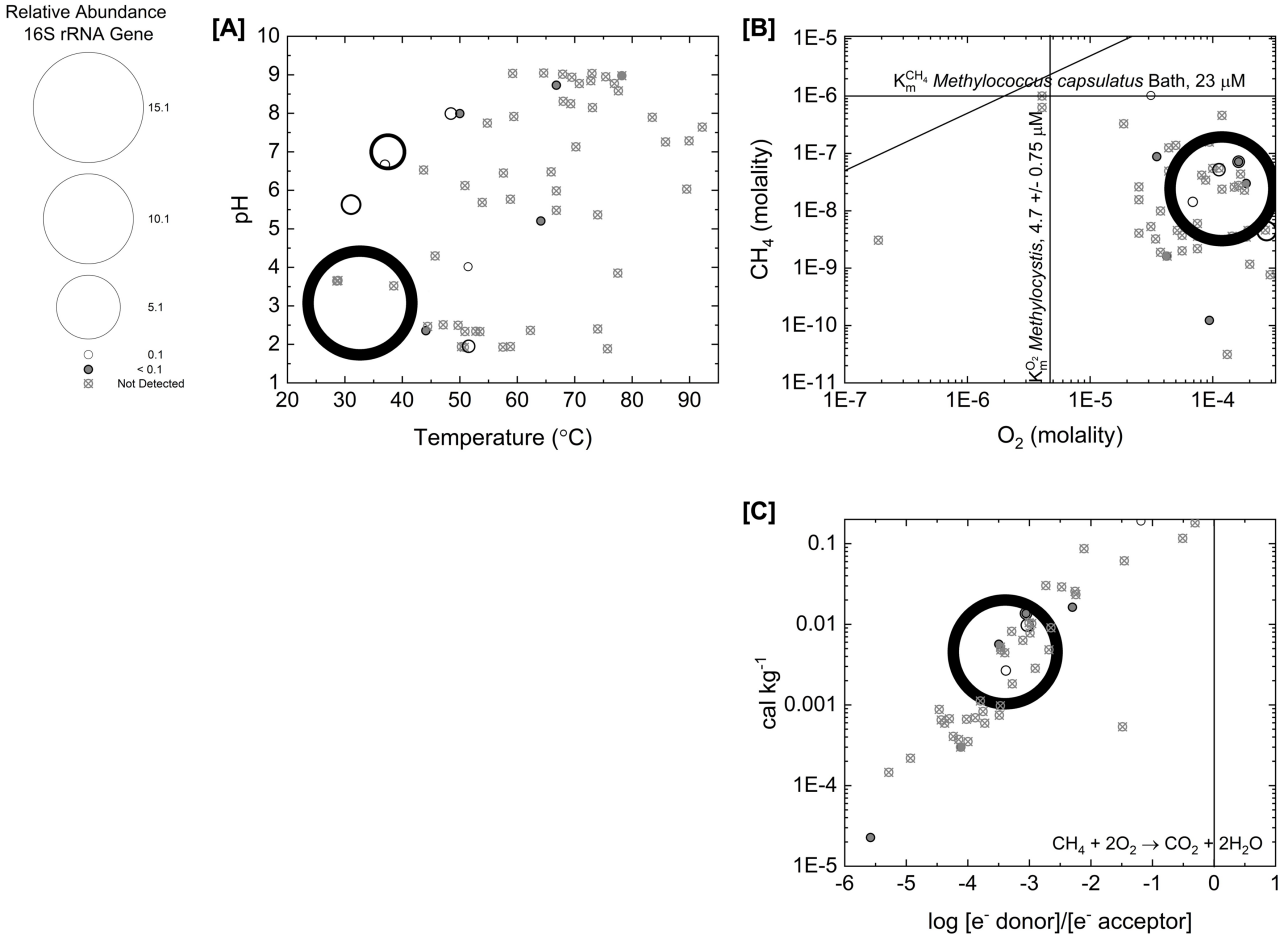
Distribution and abundance of aerobic bacterial methanotroph phylotypes as a function of pH, temperature, chemical, and energy supply data. The size of the circle scales with the summed relative abundance of the methanotroph phylotypes. Gray circles are sites where relative abundance is <1%. Circles with an “x” are sites where bacterial aerobic methanotroph phylotypes were not detected. **(A)** The distribution across pH and temperature. **(B)** The distribution across the concentrations of dissolved O_2_ and CH_4_ in hot spring fluids. Included are the *K*_m_^CH4^ of *Methylococcus capsulatus* Bath (Knief and Dunfield, 2005) and the *K*_m_^O2^ of *Methylocystis* (Gerritse and Gottschal, 1993). The line for the stoichiometric ratio of CH_4_ to O_2_ is also shown. **(C)** The distribution across energy supply for aerobic methane oxidation and the log ratio of the concentration of the electron donor (CH_4_) and electron acceptor (O_2_). The concentrations are corrected by the stoichiometry of the redox reaction and the line represents when they are stoichiometrically equal (ratio = 1).

To evaluate aerobic methanotroph distribution with respect to pH and temperature, Figure 2A illustrates the distribution of aerobic methanotroph phylotypes by plotting the summed aerobic methanotroph phylotype relative abundance as a function of pH and temperature with circle size scaled to the relative abundance. Gray circles indicate sites where relative abundance is <0.1%, and circles marked with an “x” indicate sites where no bacterial aerobic methanotrophs were detected. The observed pH distribution aligns with the optimal growth pH ranges of previously cultivated methanotrophs (Houghton et al., 2019). The highest relative abundance (16.1%, see Supplementary Table 5) of aerobic methanotrophs, associated with *Methylocystaceae* (*Alphaproteobacteria*), is at an acidic, relatively low temperature site (pH 3.0, 34.7 °C). These conditions are optimal for mesophilic methanotrophs of the family *Methylocystaceae* which are known to be acidophilic, but not thermotolerant (Bowman, 2006; Houghton et al., 2019).

To evaluate the potential influence of substrate concentration and other relevant environmental parameters on aerobic methanotroph distribution, we carried out a comparison of geochemical fluid compositions with growth parameters of previously cultivated aerobic bacterial methanotrophs summarized in Table 4 which includes previously reported half-saturation constants for activity (*K*_m_) or growth (*K*_s_) of aerobic methanotroph substrates. *Alphaproteobacteria* and *Verrucomicrobia* oxidize CH_4_, using O_2_ as an electron acceptor (Dedysh and Knief, 2018) (summary redox reaction shown in Table 2). They share the same methane oxidation pathway, in which the first step is catalyzed by the enzyme particulate methane monooxygenase (pMMO, encoded by *pmoA*). This enzyme couples the oxidation of methane to methanol with the reduction of O_2_ (Semrau et al., 2010). In these methanotrophs, methane serves as both an energy source and a carbon source (Semrau et al., 2010). Figure 2B shows the distribution of bacterial aerobic methanotroph phylotypes across measured concentrations of dissolved CH_4_ and O_2_ in hot spring fluids. For reference, the figure includes the *K*_m_^CH4^ (23 mM) of *Methylococcus capsulatus* Bath (Knief and Dunfield, 2005) and the *K*_m_^O2^ (4.7 mM) of *Methylocystis* (Gerritse and Gottschal, 1993) (see Table 4). Based on these growth parameters and the distribution shown in Figure 2B, bacterial aerobic methanotroph activity in Yellowstone hot springs is likely not limited by O_2_ availability but may be limited by the availability of CH_4_.

**Table 4:** Half-saturation constants of previously characterized methanotrophs and ammonia oxidizers.

Microbial group	Organism and reference	*K*_m_^CH4^	*K*_S_^CH4^	*K*_m_^TAN^	*K*_m_^O2^	*K*_S_^O2^	*K*_m_^NO2−^
Bacterial aerobic methanotrophs	*Methylosinus trichosporium* OB3b (Knief and Dunfield, 2005)	1					
	*Methylococcus capsulatus* Bath (Knief and Dunfield, 2005)	23					
	*Methylocystis* sp. strain MOX 1 (van Bodegom et al., 2001)		28			1.9	
	*Methylocystis* (Gerritse and Gottschal, 1993)				4.7		
Bacterial anaerobic methanotrophs	“*Candidatus* Methylomirabilis oxyfera” (Vavilin and Rytov, 2013)	120					140
Archaeal anaerobic methanotrophs	Enrichment with nitrate (Yu et al., 2017)	83					
	Enrichment with nitrate (Yu et al., 2017)	1700					
	Enrichment with sulfate (Yu et al., 2017)	>2000					
Bacterial aerobic ammonia oxidizers	*Nitrosomonas europaea* (Martens-Habbena et al., 2009)			553			
	*Nitrosomonas oceani* (Martens-Habbena et al., 2009)			101.4			
Archaeal aerobic ammonia oxidizers	“*Candidatus* Nitrosopumilus maritimus” strain SCM1 (Martens-Habbena et al., 2009)			0.134	0.134		

*K*_m_ values are the half-saturation constant for methane oxidation or ammonia oxidation activity. *K*_S_ values are the half-saturation constants for growth. *K*_m_ and *K*_s_ values are reported in μM.

To further evaluate the influence of substrate availability and energy supply on aerobic methanotroph distribution, Figure 2C plots the energy supply for aerobic methane oxidation against the log ratio of the concentration of CH_4_ to the concentration of O_2_, with concentrations corrected for the stoichiometry of the redox reaction. Aerobic methanotroph species differ in their preferred CH_4_:O_2_ tensions (Amaral and Knowles, 1994). Growth experiments by Akberdin et al. (2018) on the gammaproteobacterial species *Methylomicrobium alcaliphilum* 20Z^R^ show that CH_4_:O_2_ ratios less than −0.5 support growth. This threshold corresponds to the zero line in Figure 2C. All Yellowstone sites plot below this line, indicating that based on CH_4_:O_2_ ratios, bacterial aerobic methanotroph growth is viable in these hot springs.

**Figure 3:**
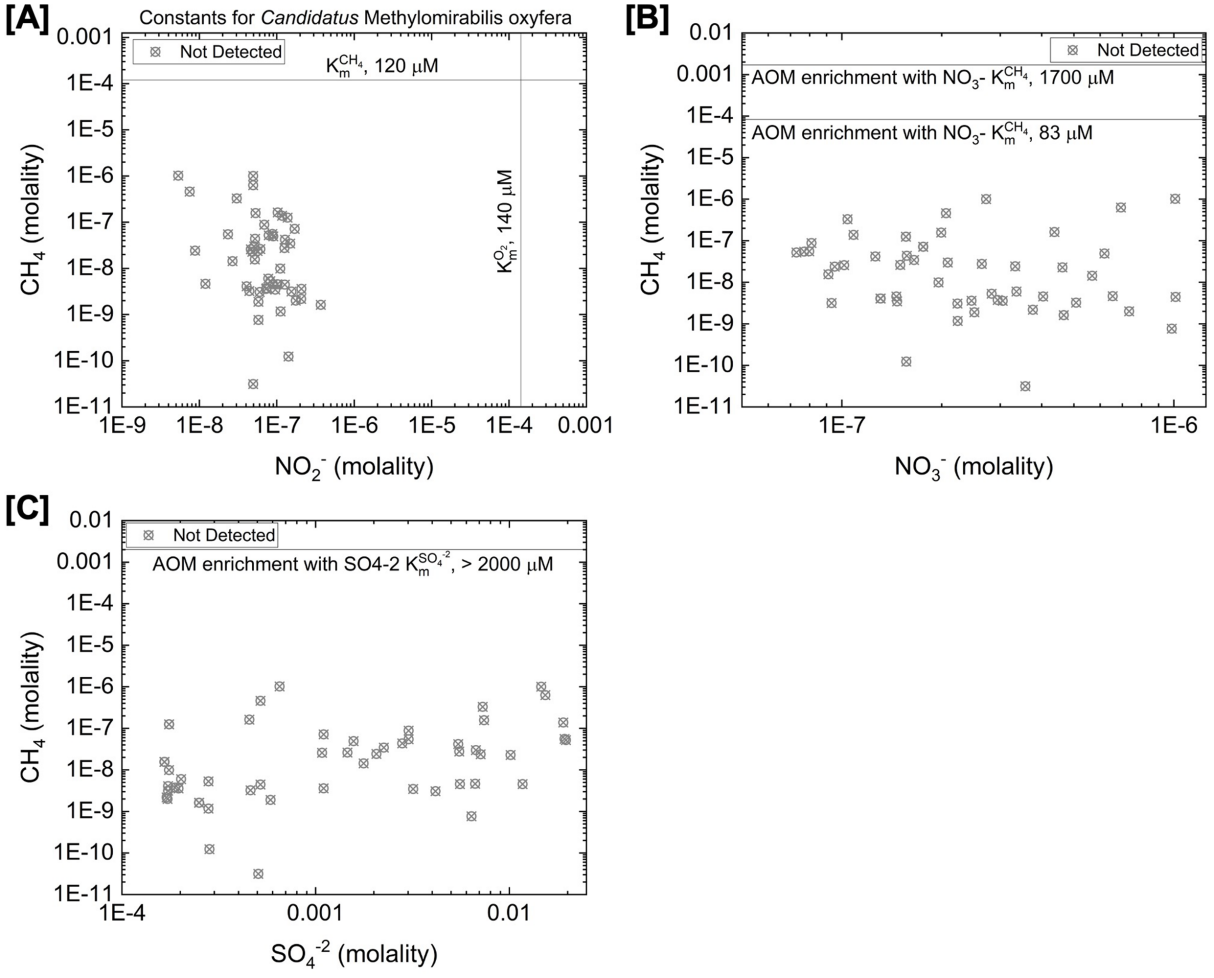
Reactant concentrations for anaerobic methane oxidation. **(A)** Concentrations of NO_2_^−^ and CH_4_ in hot spring fluids. Included are the *K*_m_NO_2_^−^ and *K*_m_^CH4^ of “Candidatus *Methylomirabilis oxyfera*” reported by Vavilin and Rytov (2013). **(B)** Concentrations of NO_3_^−^ and CH_4_ in hot spring fluids. Included is the *K*_m_^CH4^ for an anaerobic methane oxidizing consortium using NO_3_^−^ as an electron acceptor reported by Yu et al. (2017). **(C)** Concentrations of SO_4_^−2^ and CH_4_ in hot spring fluids. Included is the *K*_m_^CH4^ for an anaerobic methane oxidizing consortium using SO_4_^−2^ as an electron acceptor reported by Yu et al. (2017).

No anaerobic archaeal methanotrophs were detected through our 16S rRNA gene sequencing approach although it is important to note our universal prokaryote primer set may not capture all archaeal diversity. To explore possible environmental explanations for their lack of detection, we evaluate substrate concentrations for anaerobic methane oxidation within the context of previously characterized growth parameters of anaerobic methanotrophs reviewed in Table 4. Anaerobic methane oxidation occurs via several distinct pathways. Within the bacterial phylum NC10, the bacterial methanotroph “*Candidatus* Methylomirabilis oxyfera” has been shown to couple methane oxidation to nitrite reduction (Vavilin and Rytov, 2013). This organism uses the same core pathway as aerobic methanotrophs but generates O_2_ internally via nitrite reduction to drive methane oxidation (Wu et al., 2011). Archaeal anaerobic methanotrophs (ANME) can couple methane oxidation to the reduction of nitrate or sulfate, performing reverse methanogenesis under methane-rich conditions (Girguis et al., 2005; Thauer, 2011; Ding et al., 2016; Yu et al., 2017). In these systems, ANME transfer electrons derived from methane to partner microorganisms capable of reducing NO_3_^−^ or SO_4_^2−^ (Yu et al., 2017). Figure 3 shows the concentrations of electron acceptors for anaerobic methanotrophic metabolisms plotted against CH_4_ concentration. The *K*_m_^CH4^ values for the anaerobic methanotroph “*Candidatus* Methylomirabilis oxyfera” and for ANME consortia grown with NO_3_^−^ or SO_4_^2−^ as electron acceptors are approximately two to four orders of magnitude higher than CH_4_ concentrations measured in Yellowstone hot spring fluids. This comparison suggests that the lack of detection of anaerobic methanotrophs could be explained by limited CH_4_ availability.

Given the previous detection of archaeal ammonia oxidizers in Yellowstone hot springs (Weeks et al., 2023; Weeks, 2025) we explore the potential for this microbial group to influence methane oxidation by evaluating their 16S rRNA gene phylotype distribution with respect to previously characterized growth parameters summarized in Table 5. The enzyme used for the first step of ammonia oxidation—ammonia monooxygenase (AMO, encoded by *amoA*)—is structurally and functionally similar to pMMO, and phylogenetic evidence indicates that *pmoA* and *amoA* share a common ancestor (Khadka et al., 2018). Experimental studies have shown that bacterial ammonia oxidizer activity can be inhibited by methane and, while these organisms cannot grow using methane as their sole substrate, they exhibit measurable methane oxidation activity (Jones and Morita, 1983; Ward, 1990). More recently, work in volcanic soils demonstrated that archaeal ammonia oxidizers can incorporate methane into biomass (Daebeler et al., 2014). Table 5 summarizes inhibition constants (*K*ᵢ) for CH_4_ inhibition of bacterial ammonia oxidation and NH_3_ inhibition of methane oxidation. The *K*_i_^CH4^ reported in Table 5 is from a study on the bacterial ammonia oxidizer *Nitrosomonas europaea* (Suzuki et al., 1974). Aerobic bacterial methanotrophs are also capable of oxidizing NH_3_ to NO_2_^−^ (Nyerges and Stein, 2009), and the *K*_m_^NH3^ values for selected methanotrophs are also included in Table 5. Together, previous findings suggest that the actions of both bacterial and archaeal ammonia oxidizers could result in methane sinks.

**Table 5:** Activity and inhibition constants for CH_4_ and NH_3_ competition.

Microbial group	Organism and reference	*K*_I_^CH4^	*K*_m_^NH3^	*K*_I_^NH3^
Bacterial aerobic methanotrophs	*Methylocystis* sp. strain ATCC 49242 (Nyerges and Stein, 2009)		560*	
	*Methylocystis* sp. strain ATCC 49242 + 5,000 ppm CH4 (Nyerges and Stein, 2009)		1950*	
	*Methylomicrobium album* ATCC 33003 (Nyerges and Stein, 2009)		200*	
	*Methylomicrobium album* ATCC 33003 + 5,000 ppm CH4 (Nyerges and Stein, 2009)		490*	
	*Methylosinus trichosporium* OB3b pH 6.5 (O’Neill and Wilkinson, 1977)		7.4*	
	*Methylosinus trichosporium* OB3b pH 7.5 (O’Neill and Wilkinson, 1977)		10.4*	
	*Methylosinus trichosporium* OB3b pH 6 (O’Neill and Wilkinson, 1977)			9.8
	*Methylosinus trichosporium* OB3b pH 8 (O’Neill and Wilkinson, 1977)			11.2
Bacterial aerobic ammonia oxidizers	*Nitrosomonas europaea* (Suzuki et al., 1974)	50^a^		

*Values may be total ammonia nitrogen as methods for chemical speciation of NH_3_ were not described.

^a^Experiments done on cell extract.

Review of ammonia half-saturation constants for ammonia oxidation activity by methanotrophs (*K*_m_^NH3^), ammonia inhibition constant for methane oxidation activity (*K*_m_^NH3^), and methane inhibition constant for ammonia oxidation activity (*K*_I_^CH4^). Values are in μM.

**Figure 4:**
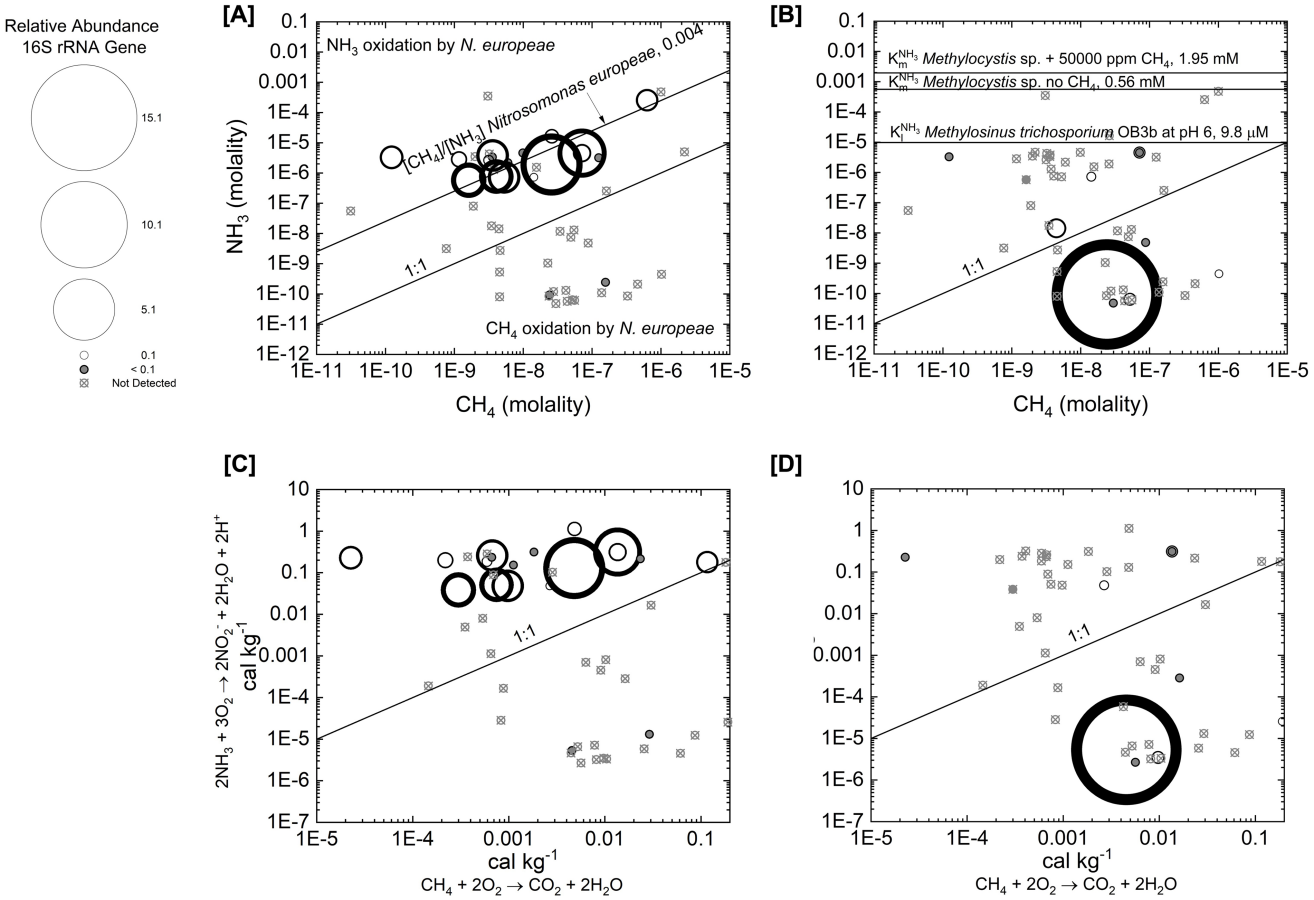
Chemical interactions between NH_3_ and CH_4_. **(A)** The distribution of bacterial aerobic methanotroph phylotypes across NH_3_ concentration (estimated from total ammonia nitrogen) and CH_4_ molality. Included is the K_I_^NH3^ for *Methylosinus trichosporium* reported by O’Neill and Wilkinson (1977) and *K*_m_^NH3^ for *Methylocystis* with no CH_4_ and with 50,000 ppm CH_4_ reported by Nyerges and Stein (2009). Also drawn is the 1:1 line for the activity of NH_3_ to the concentration of CH_4_. **(B)** The distribution of archaeal ammonia oxidizer phylotypes across NH_3_ and CH_4_ concentration. Included is the *k*_cat_/*K*_m_ of the bacterial ammonia oxidizer *Nitrosomonas europaea* for either NH_3_ oxidation ([CH_4_]/[NH_3_] < 0.004) or CH_4_ oxidation ([CH_4_]/[NH_3_] > 0.004) (Ward, 1990). **(C)** The distribution of bacterial aerobic methanotroph phylotypes across energy supply for aerobic methane oxidation and aerobic ammonia oxidation. **(D)** The distribution of archaeal ammonia oxidizer phylotypes across energy supply for aerobic methane oxidation and aerobic ammonia oxidation.

To investigate the potential for methane oxidation by archaeal ammonia oxidizers detected in this study, Figure 4A shows the distribution of archaeal ammonia oxidizer phylotypes as a function of NH_3_ concentration and CH_4_ concentration, with a 1:1 line indicating equal molality of CH_4_ and NH_3_ activity. Also shown is the [CH_4_]:[NH_3_] ratio corresponding to the observed *k*_cat_/*K*_m_ of the bacterial ammonia oxidizer *Nitrosomonas europaea* for either ammonia oxidation ([CH_4_]/[NH_3_] < 0.004) or CH_4_ oxidation ([CH_4_]/[NH_3_] > 0.004) (Ward, 1990). Archaeal ammonia oxidizer phylotypes were detected primarily at sites that are NH_3_-rich relative to CH_4_ (below the 1:1 line). Notably, the highest relative abundances occurred at sites lying directly on the *k*_cat_/*K*_m_ threshold line. Few sites fell above this threshold, which corresponds to the most NH_3_-rich conditions relative to CH_4_. These patterns suggest that the relative concentration of CH_4_ to NH_3_ concentration exerts a strong constraint on archaeal ammonia oxidizer activity and growth in Yellowstone hot springs.

To investigate potential ammonia oxidation by aerobic bacterial methanotrophs. Figure 4B shows the distribution of bacterial aerobic methanotroph phylotypes as a function of NH_3_ and CH_4_ concentration. Also indicated are the *K*_i_^NH3^ of *Methylosinus trichosporium* (O’Neill and Wilkinson, 1977), the *K*_m_^NH3^ of *Methylomicrobium album* ATCC and *Methylocystis* sp. strain ATCC 49242 (Nyerges and Stein, 2009), and the 1:1 line representing equal CH_4_ concentration and NH_3_ concentration. Most sites fall below the *K*_i_^NH3^ and all sites fall below the *K*_m_^NH3^ values with respect to NH_3_ concentration. These patterns suggest that NH_3_ concentration is not high enough to fully inhibit aerobic methane oxidation by methanotrophs and that NH_3_ oxidation by aerobic bacterial methanotrophs is unlikely. However, methanotroph phylotypes are generally most abundant at sites that are relatively NH_3_-poor and CH_4_-rich, with the highest relative abundance occurring at a site where CH_4_ exceeds NH_3_ activity (above the 1:1 line). This pattern indicates that NH_3_ availability may still constrain aerobic methanotroph activity and growth in Yellowstone hot springs.

The distributions of archaeal ammonia oxidizer phylotypes (Figure 4C) and bacterial aerobic methanotroph phylotypes (Figure 4D) were evaluated relative to the energy yields of aerobic CH_4_ oxidation and NH_3_ oxidation. Although aerobic methanotroph phylotypes were detected at relatively few sites, their highest relative abundance occurred at a site more energy-rich for aerobic CH_4_ oxidation than for NH_3_ oxidation (above the 1:1 line in Figure 4D). In contrast, archaeal ammonia oxidizer phylotypes were most abundant at sites more energy-rich for aerobic NH_3_ oxidation than CH_4_ oxidation. These patterns suggest that competitive chemical interactions between NH_3_, CH_4_, and monooxygenases may be driven in part by the energy supplies of their respective redox reactions.

The sparse distribution of aerobic methanotrophs in Yellowstone hot springs based on 16S rRNA gene phylotypes is further supported by a meta-analysis we conducted on shotgun metagenome assembled genomes from 34 springs reported by Colman et al. (2024). The hot springs studied by Colman et al. (2024) do not overlap with the sites in our study and therefore further expand the molecular survey for the functional potential for biological methane oxidation. We surveyed the presence of genes encoding monooxygenase enzymes including particulate methane monooxygenase, soluble methane monooxygenase, ammonia monooxygenase, and alkane monooxygenases described in Wang et al. (2023) summarized in Supplementary Table 7. Genes encoding particulate methane monooxygenase were not detected and in only two hot springs (pH 7.2, 79.4 °C; pH 7.5, 74.8 °C) we detected ammonia monooxygenases within genomes taxonomically classified as belonging to the genus *Nitrosocaldus*. Soluble methane monooxygenase was detected in several systems with pH values ranging from 2.3 to 9.0 and temperatures 61.9 °C to 92.8 °C (Supplementary Table 7), although none of the MAGs that contain genes encoding soluble methane monooxygenases are known aerobic methanotrophs, which lowers our confidence in their potential function as methane monooxygenases. We also investigated functional potential for anaerobic methane oxidation and found there is little evidence for genes encoding divergent methyl co-enzyme M reductase used by anaerobic methanotrophs. Those that we detected are likely associated with methanol-dependent methanogenesis described by Lynes et al. (2023).

Taken together, our models of substrate availability, energy supply, and molecular distribution analyses indicate that both aerobic methane oxidation by bacterial methanotrophs and aerobic ammonia oxidation by archaeal ammonia oxidizers are thermodynamically and ecologically viable in Yellowstone hot springs. Yet, NH_3_ availability likely constrains aerobic methanotroph activity and growth to sites where CH_4_ is relatively enriched. Conversely, CH_4_ concentrations at many sites are sufficiently high—relative to NH_3_—to permit CH_4_ oxidation by at least the bacterial ammonia oxidizer *Nitrosomonas europaea*, and plausibly by archaeal ammonia oxidizers as well. Strikingly, the strongest signal of archaeal ammonia oxidizer phylotypes occurs at the most NH_3_-rich sites relative to CH_4_, suggesting a partitioning of their distribution based on competitive inhibition of ammonia oxidation by CH_4_. This integration of chemical energy supplies with microbial community structure highlights a dynamic interplay between substrate availability, enzyme specificity, and niche partitioning, revealing that ammonia-oxidizing archaea could contribute to methane oxidation under extreme geothermal conditions.

### 3.2 Biological methane oxidation rates in YNP hot springs

Geochemical analysis of hot spring fluids, 16S rRNA gene composition of hot spring sediments, and growth parameters of previously cultivated aerobic methanotrophs indicate that biological aerobic methane oxidation is likely to occur in Yellowstone hot spring ecosystems, though it may be limited by CH_4_ availability and chemically inhibited by NH_3_. As seen previously by Weeks (2025), and in this dataset, geochemical and sequencing data also suggest that Yellowstone hot springs are suitable habitats for archaeal ammonia oxidizers, with potential for these organisms to promiscuously oxidize methane.

**Figure 5:**
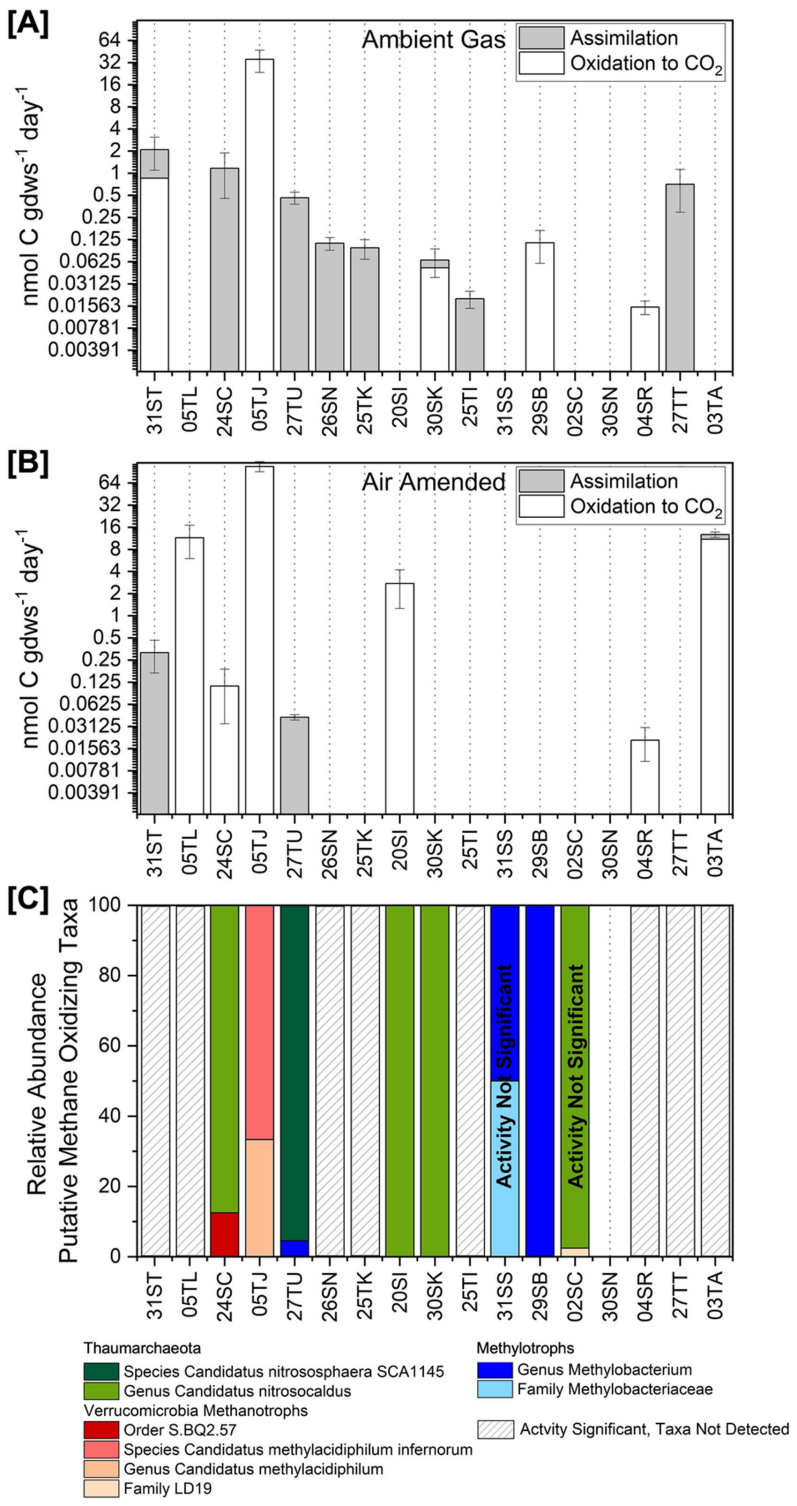
Biological rates of methane oxidation. Rates for ambient gas and air-amended conditions are shown as well as the composition of putative methane oxidizing taxa at each experimental site; sites are ordered by increasing temperature from left to right. Bars filled in gray are the rate of CH_4_ assimilation into biomass; white bars are rates of oxidation to CO_2_. Error bars are the standard deviation of the total MOR. **(A)** Measured rates of methane oxidation in ambient gas experiments. **(B)** Measured rates of methane oxidation in air-amended experiments. **(C)** The composition of putative methane oxidizing taxa detected at the experimental sites. The relative abundance is percent composition of the total putative methane oxidizing taxa detected and not relative abundance within the whole microbiome. Overall, taxa detected at experimental sites make up <1% of the microbiome.

To assess whether biological methane oxidation is actively occurring, *in situ* microcosm assays were performed at 17 of the 61 sites where 16S rRNA gene sequencing was conducted, using the conversion of ^14^CH_4_ to ^14^CO_2_ and incorporation of ^14^C into biomass as indicators of activity. These sites spanned a range of pH values (1.9–9.0) and temperatures (38.5–89.9 °C) and included locations both with and without known aerobic methanotroph sequences. Two experimental conditions were tested: one maintaining ambient hot spring gas composition by setting up microcosms with a N_2_ headspace, and another with air to act as an O_2_ amendment (see Methods). To account for possible abiotic methane oxidation, biological assays were compared to chemical kill controls (6 M NaOH additions, see Methods). All experiments and controls were conducted in triplicate. Rates were considered biological when significantly higher than kill controls (*t*-test, *p* < 0.05). Rates of CH_4_ oxidation to CO_2_, assimilation into biomass, and their sum for each experimental condition are reported in Table 6. Figure 5 summarizes these rates across sites for ambient gas (Figure 5A) and air-amended (Figure 5B) treatments, while Figure 5C shows the composition of putative methane-oxidizing taxa at each experimental site.

**Table 6:** Measured biological rates of methane oxidation.

Sample ID	Figure ID	Temperature (°C)	pH	Ambient gas, CO_2_	Ambient gas, CO_2_	Ambient gas, assimilation	Ambient gas, assimilation	Ambient gas, total	Ambient gas, total
					Standard deviation		Standard deviation		Standard deviation
140731ST	31ST	38.5	3.5	0.85	0.459	1.24	0.89	2.09	1.00
140805TL	05TL	45.7	4.3						
140724SC	24SC	50.0	8.0			1.17	0.71	1.17	0.71
140805TJ	05TJ	52.9	4.0	35.35	11.887	0.00	0.00	35.35	11.89
140727TU	27TU	57.6	6.4			0.46	0.09	0.46	0.09
140726SN	26SN	65.9	6.5			0.11	0.02	0.11	0.02
140725TK	25TK	66.8	6.0			0.10	0.03	0.10	0.03
140730SI	20SI	68.0	8.3						
140730SK	30SK	73.1	8.1	0.05	0.027	0.01	0.01	0.07	0.03
140725TI	25TI	74.0	5.4			0.02	0.01	0.02	0.01
140731SS	31SS	75.7	1.9						
140729SB	29SB	78.1	2.2	0.11	0.053			0.11	0.05
140802SC	02SC	78.2	9.0						
140730SN	30SN	83.5	7.9						
140804SR	04SR	85.8	7.3	0.02	0.003	0.00	0.00	0.02	0.00
140727TT	27TT	89.5	6.0			0.71	0.41	0.71	0.41
140803TA	03TA	89.9	7.3						

Sample ID	Figure ID	Temperature (°C)	pH	Air amended, CO_2_	Air Amended, CO_2_	Air amended, assimilation	Air amended, assimilation	Air amended, total	Air amended, total
					Standard deviation		Standard deviation		Standard deviation
140731ST	31ST	38.5	3.5			0.31	0.149	0.31	0.15
140805TL	05TL	45.7	4.3	11.52	5.534			11.52	5.53
140724SC	24SC	50.0	8.0	0.11	0.077			0.11	0.08
140805TJ	05TJ	52.9	4.0	107.19	16.754			107.19	16.75
140727TU	27TU	57.6	6.4			0.04	0.004	0.04	0.00
140726SN	26SN	65.9	6.5						
140725TK	25TK	66.8	6.0						
140730SI	20SI	68.0	8.3	2.74	1.484			2.74	1.48
140730SK	30SK	73.1	8.1						
140725TI	25TI	74.0	5.4						
140731SS	31SS	75.7	1.9						
140729SB	29SB	78.1	2.2						
140802SC	02SC	78.2	9.0						
140730SN	30SN	83.5	7.9						
140804SR	04SR	85.8	7.3	0.02	0.010			0.02	0.01
140727TT	27TT	89.5	6.0						
140803TA	03TA	89.9	7.3	10.98	0.749	1.71	0.763	12.69	1.07

Values are in nmol C gdws^−1^·day^−1^. Experimental rates are defined as biological when biological activities are significantly greater than kill controls (*t*-test *p*-value is < 0.05).

Significant rates of biological methane oxidation in comparison to killed controls were detected at 14 of the 17 experimental sites. CH_4_ oxidation attributed to biological activity was detected in 11 of the ambient gas experiments (Figure 5A) and in 8 of the air-amended experiments (Figure 5B), with 5 sites showing activity under both conditions. Differences in the occurrence of biological methane oxidation between treatments suggest that methane-oxidizing organisms in these systems vary in their O_2_ preferences.

Assimilation of ^14^CH_4_ into biomass was observed more frequently in ambient gas experiments (8 of 11) than in air-amended experiments (3 of 8). Notably, at 6 of the 8 sites where assimilation under ambient gas conditions occurred, no oxidation of ^14^CH_4_ to ^14^CO_2_ was detected. This pattern was also observed at 2 of the 3 sites with assimilation under air-amended conditions. In cultivated bacterial aerobic methanotrophs, complete oxidation of CH_4_ to CO_2_ provides electrons as NADH to the electron transport chain for energy generation (Semrau et al., 2010). Therefore, at sites where only ^14^CH_4_ assimilation was detected, it is possible that methane-oxidizing organisms are relying on alternative energy sources.

Bacterial aerobic methanotroph phylotypes were detected at 2 of the 14 sites where significant MORs were measured (Figure 5C), and it is likely that the observed activity at these sites was carried out by the detected taxa. The highest total MOR, 107 nmol C gdws^−1^·day^−1^, occurred at a spring (pH 4.0, 53 °C) where only *Verrucomicrobia* methanotroph phylotypes were detected. These pH and temperature conditions are comparable to a hot spring in Kamchatka, Russia (pH 3, 52 °C), where the *Verrucomicrobia* methanotroph Kam1 was isolated (Islam et al., 2008).

Archaeal ammonia oxidizer phylotypes (“*Candidatus* Nitrososphaera SCA1145” and “*Candidatus* nitrosocaldus”) were detected at 4 of the 14 sites with significant MORs. The highest rate from these sites (2.7 nmol C gdws^−1^·day^−1^) was measured at pH 8.3 and 68 °C, where activity was observed only in the air-amended experiment, with no ^14^CH_4_ assimilation into biomass. Methane oxidation associated with archaeal ammonia oxidizers was also detected (0.1 nmol C gdws^−1^·day^−1^) at a high temperature, acidic site (pH 2.2, 78.1 °C) where phylotypes of *Methylobacterium* (*Alphaproteobacteria*) were detected. *Methylobacterium* are facultative methylotrophs that can oxidize methanol to formaldehyde for downstream carbon assimilation (Schrader et al., 2009). At this site (140727TU in Figure 5) only ^14^CH_4_ assimilation was detected, which suggests the putative archaeal ammonia oxidizing taxa (“*Candidatus* nitrosocaldus”) may have promiscuously oxidized methane to methanol and putative *Methylobacterium* may have assimilated the ^14^C methanol. This is not the first observation of carbon assimilation associated with methane oxidation by ammonia oxidizers. In culture studies, bacterial ammonia oxidizers can oxidize CH_4_ to CO_2_ and assimilate CH_4_ when CH_4_ inhibits ammonia oxidation (Jones and Morita, 1983). In an environmental stable isotope probe study with ^13^CH_4_ amendments, archaeal ammonia oxidizers incorporated methane-derived carbon into DNA (Daebeler et al., 2014). These findings suggest that the activity observed here is likely associated with the detected archaeal ammonia oxidizer phylotypes.

At eight sites, significant MORs were measured despite no detection of known methane-oxidizing taxa. In using such a sensitive technique (tracing radioactive C) it is possible that we have captured slow microbial processes for methane oxidation that have yet to be characterized. Remarkably, methane oxidation under air-amended conditions was observed at a site with a temperature of 89.9 °C (site 140803TA)—17.9 °C above the current upper temperature limit for methanotrophs in culture and 14.9 °C higher than previous assessments of methanotrophic activity in New Zealand hot springs (Houghton et al., 2023). The discovery of novel methanotrophic lineages in recent decades, including members of *Verrucomicrobia* and NC10 (Pol et al., 2007; Vavilin and Rytov, 2013), highlight the potential for uncharacterized methanotroph lineages to carry out methanotrophy at such extreme conditions.

When comparing measured rates to those reported for geothermal soils in New Zealand by Houghton et al. (2023), methane oxidation in Yellowstone hot springs is substantially slower. In our study, rates ranged from 0.02 to 107.2 nanomol CH_4_ gdws^−1^·day^−1^, whereas rates in New Zealand geothermal soils ranged from 0.5 to 17.4 micromol CH_4_ gdws^−1^·day^−1^. This difference likely reflects variation in substrate availability. Although we cannot directly compare the geochemical characteristics of the geothermal soils reported by Houghton et al. (2023) with those of Yellowstone hot springs, CH_4(aq)_ concentrations in hot springs of geothermal regions of New Zealand range from 11 nanomolar to 41 micromolar (Power et al., 2018; 1000springs.org.nz), whereas in our study they range from 31 picomolar to 1.0 micromolar. On average, CH_4_ concentrations are three orders of magnitude higher in New Zealand hot springs than in those we surveyed in Yellowstone. Furthermore, the *K*_m_^CH4^ and *K*_s_^CH4^ of characterized aerobic methanotrophs (see Table 4) fall within the methane concentration range of New Zealand hot springs, whereas Yellowstone hot spring methane concentrations do not. Together, these observations suggest that New Zealand geothermal systems likely emit more methane overall and may better support biological methane oxidation.

Overall, our results reveal that biological methane oxidation is not only possible but is likely active and widespread in Yellowstone hot springs, spanning a broad range of temperatures and fluid chemistries. Activity assays combined with 16S rRNA gene sequencing link portions of this activity to *Verrucomicrobia* and *Alphaproteobacteria* methanotrophs, while also pointing to a possible role for archaeal ammonia oxidizers—organisms not traditionally recognized for methane oxidation. Perhaps most intriguingly, significant activity was detected at multiple sites where no known methane- or ammonia-oxidizing phylotypes were present, including some of the hottest continental environments ever associated with methane oxidation. These findings point to the existence of thermophilic methanotrophs from uncharacterized microbial lineages, expanding the known limits and diversity of life capable of oxidizing methane.

### 3.3 Possible drivers of observed methane oxidation rates in YNP hot springs

**Figure 6:**
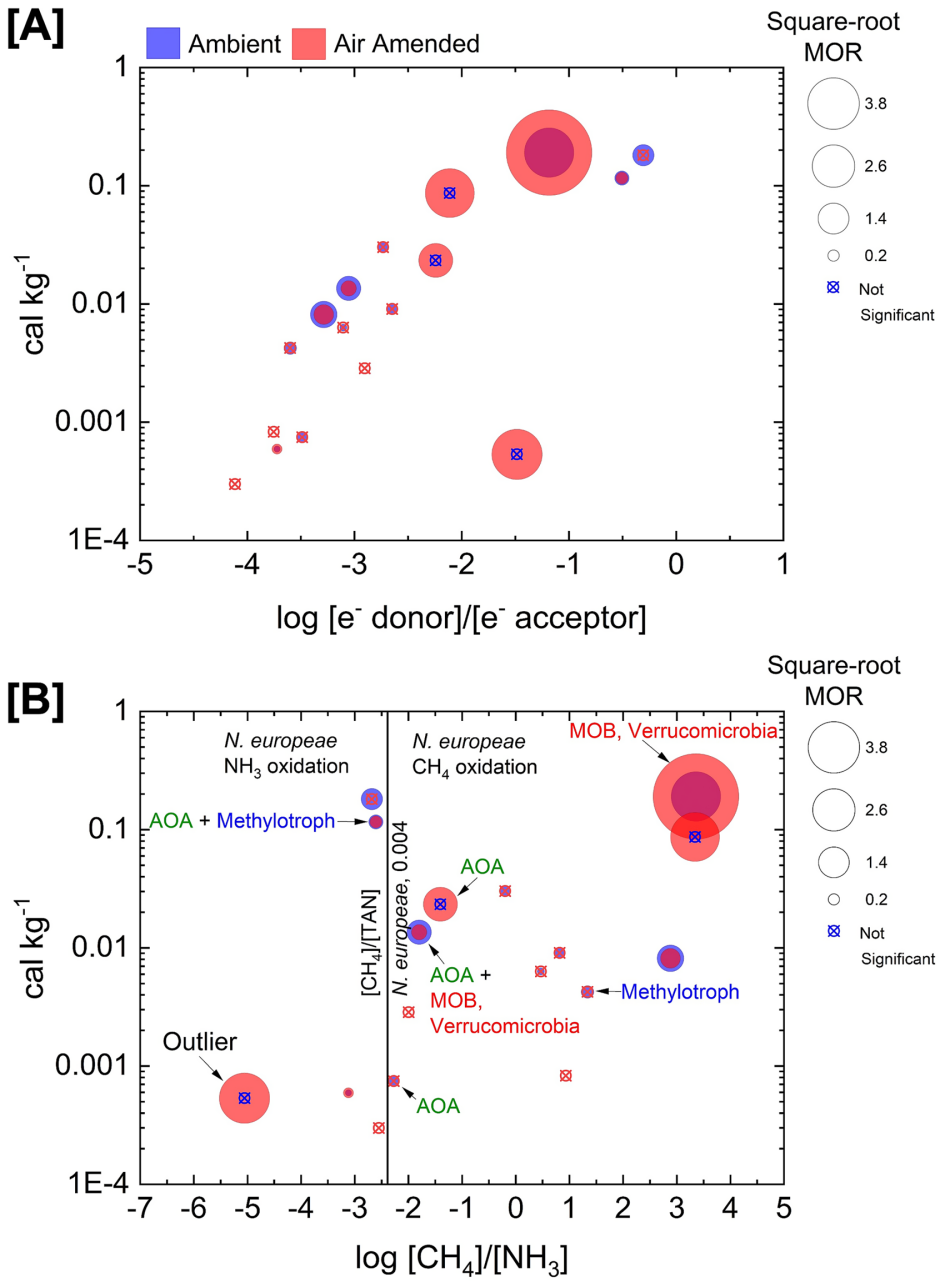
Multivariate view of rates. The size of the circle scales with the square-root of the measured MOR. Rates for both the ambient gas (blue circles) and air-amended microcosms (red circles) are shown. **(A)** The measured rates of methane oxidation plotted as a function of the log ratio of the reactants and energy supply for aerobic methane oxidation. **(B)** The measured rates are plotted as a function of the log ratio of the concentrations of CH_4_ and NH_3_ and energy supply for aerobic methane oxidation. Sites where rates were measured and methane oxidizing taxa were detected are labeled with the detected taxa. Archaeal ammonia oxidizers are AOA and bacterial methanotrophs are MOB. Red MOB are sites where *Verrucomicrobia* methanotrophs were detected. Blue MOB are sites where *Alphaproteobacteria* were detected. Also shown is the [CH_4_]:[NH_3_] ratio which corresponds to the observed *k*_cat_/*K*_m_ of the bacterial ammonia oxidizer *Nitrosomonas europaea* for either NH_3_ oxidation ([CH_4_]/[NH_3_] < 0.004) or CH_4_ oxidation ([CH4]/[NH_3_] > 0.004) (Ward, 1990). The site labeled “Outlier” is a site with a relatively high rate of CH_4_ oxidation despite have a [CH_4_] to [NH_3_] ratio <0.004.

To explore factors that may influence the observed biological MORs, we examined rates in the context of two fundamental controls: the stoichiometry-corrected log ratio of CH_4_ to O_2_ concentrations on the *x*-axis, and the calculated energy supply for aerobic methane oxidation on the *y*-axis in Figure 6A. Rates from both experimental conditions are shown—blue for ambient gas incubations and red for air-amended incubations—with “*x*” marks indicating sites lacking significant activity. This integrative view reveals a clear trend: the most CH_4_-rich sites relative to O_2_ tend to sustain the highest MORs, and the single highest rate aligns with the largest calculated energy supply. Yet the picture is not purely linear—several energy-rich sites display low rates, suggesting that high thermodynamic potential alone does not guarantee high biological activity. At one such high-energy, low-rate site, archaeal ammonia oxidizer phylotypes were present, hinting that chemical inhibition by ammonia, rather than energy limitation, may be the key constraint.

To interrogate this hypothesis we plot MORs against the stoichiometry-corrected log ratio of CH_4_ to NH_3_ concentration, again with energy supply on the *y*-axis in Figure 6B. Here, the reference line for the bacterial ammonia oxidizer *Nitrosomonas europaea* serves as a benchmark for competitive potential between methane and ammonia oxidation. Strikingly, the high-energy, low-rate sites from Figure 6A cluster in the NH_3_-rich region of Figure 6B and are dominated by archaeal ammonia oxidizers (AOA). In contrast, the CH_4_-rich, NH_3_-poor site yielding the highest rate is dominated by bacterial aerobic methanotrophs (MOB). This distribution suggests a competitive threshold: when NH_3_ availability is high relative to CH_4_, methane oxidation may be suppressed both through direct enzyme-level competition for NH_3_ by ammonia monooxygenase and through ecological dominance of organisms that are not methane specialists. In Yellowstone hot springs, these interactions appear to override the advantages conferred by abundant energy supply, suggesting that the landscape of biological methane oxidation may be shaped not only by thermodynamics but by physiological constraints like chemical inhibition.

### 3.4 Methane oxidation associated with a hydrothermal hydrocarbon degrading community

Alkane oxidizers (alkanotrophs) which aerobically oxidize short-chain alkanes such as ethane, butane, and propane for energy (Osborne and Haritos, 2019), may be another sink for methane in hot springs. The enzymes involved in alkane oxidation are structurally and functionally similar to soluble methane monooxygenase (sMMO), an alternative to pMMO used by some aerobic methanotrophs (Osborne and Haritos, 2019). Phylogenetic analyses suggest sMMO evolved from butane monooxygenase (Osborne and Haritos, 2019). Notably, organisms such as *Thauera butanivorans*, which can oxidize butane, also exhibit methane oxidation activity, though they cannot grow solely on methane (Cooley et al., 2009). This indicates that alkanotrophs may represent an additional methane oxidation pathway in some environments.

In both Figures 6A,B, a pronounced outlier is evident: a high rate of methane oxidation in the air-amended experiments was observed at pH 7.3 and 89.9 °C, despite the site fluid chemistry yielding comparatively low energy supply for methane oxidation. This location also exhibited the highest NH_3_-to-CH_4_ ratio among all sites. The site, located at Calcite Springs in Yellowstone, has previously been reported to contain detectable levels of hydrocarbons (Clifton et al., 1990; Nye et al., 2020). It is possible that, due to homology between enzymes used by alkanotrophs and methanotrophs, methane oxidation activity at this site is associated with organisms that oxidize C-2 and larger alkanes.

To further explore the potential for microbial hydrocarbon degradation in this system, we performed shotgun metagenomic sequencing on DNA extracted from sediment collected at the same site used for the methane oxidation experiments and at the same time. MAGs were annotated with a specific focus on identifying monooxygenase family enzymes following guidance from Chamy and Rosenkranz (2013). The resulting amino acid sequences were queried against the NCBI protein BLAST database, and detailed results are provided in Table 7. While no canonical particulate or soluble methane monooxygenases were detected, we identified potential nitronate monooxygenase (pNMO) genes in three MAGs (Bin6: 99.7% complete, 0% contamination; Bin8: 91.9% complete, 2% contamination; and Bin12: 77.2% complete, 0.8% contamination). pNMO catalyzes the oxidative denitrification of alkyl nitronates using molecular oxygen (Gadda and Francis, 2010). Given the functional similarity between pNMO and alkane monooxygenases, it is plausible that pNMO could exhibit promiscuous activity toward methane oxidation, representing a potentially overlooked enzymatic pathway in hydrothermal and hydrocarbon-rich environments. Although the enzymatic function of few proteins encoded by genes annotated as nitronate monooxygenase has been assessed, studies suggest that they may exhibit oxidase activity (Ball et al., 2016), which is further supported by the homology of pNMO with other monooxygenases (Wang et al., 2023). However, we acknowledge the limitations of sequence-based annotations in confidently inferring protein function.

**Table 7:** Summary of monooxygenase genes in a high temperature, hydrocarbon rich hot spring.

Bin #		Bin9	Bin9	Bin9	Bin12	Bin6	Bin8
Bin GTDB-k taxonomy	Order	Syntrophobacterales	Syntrophobacterales	Syntrophobacterales	Hydrogenothermales	Dictyoglomales	Deferribacterales
	Family or genus	F: Thermodesulforhabdaceae	F: Thermodesulforhabdaceae	F: Thermodesulforhabdaceae	G: Sulfurihydrogenibium	G: OB1-4	G: Calditerrivibrio
% Completeness		97.58	97.58	97.58	77.82	99.66	91.86
% Contamination		0.81	0.81	0.81	0.81	0	1.97
Monooxygenase, BLASTp annotation	Annotation	FAD-dependent oxidoreductase	CoB-CoM heterodisulfide reductase iron-sulfur subunit A family protein	Antibiotic biosynthesis monooxygenase family protein	Nitronate monooxygenase family protein	Nitronate monooxygenase	Nitronate monooxygenase
	Percent Identity	72.92%	73.66%	69.15%	98.94%	87.61%	85.19%
	Order	Syntrophobacterales	Syntrophobacterales	Syntrophobacterales	Aquificales	Dictyoglomales	Deferribacterales
	Genus	*Desulfacinum*	*Desulfacinum*	*Thermodesulforhabdus*	*Sulfurihydrogenibium*	*Dictyoglomus*	*Calditerrivibrio*
Metabolism		Sulfate-reducing bacteria	Sulfate-reducing bacteria	Sulfate-reducing bacteria	Sulfur-oxidizing bacteria	Carbohydrate fermenter	Nitrate-reducing bacteria
Environment		Hydrocarbon system: petroleum reservoir	Hydrocarbon system: petroleum reservoir	Hydrocarbon system: oil field	Yellowstone National Park	Alkaline hot spring Japan	Terrestrial hot spring Japan
References		Rees et al. (1995)	Rees et al. (1995)	Beeder et al. (1995)	Nakagawa et al. (2005), Takai et al. (2003)	Saiki et al. (1985)	Iino et al. (2008)

A notable taxon recovered from this system belongs to the order *Syntrophobacterales* (Bin9; 97% completeness, 0.81% contamination). All monooxygenase-encoding amino acid sequences from this bin showed high similarity to hydrocarbon-degrading microorganisms previously reported in the literature, including *Desulfacinum infernum* (73% amino acid identity; Rees et al., 1995) and *Thermodesulforhabdus norvegica* (69% identity; Beeder et al., 1995). Both species are thermophilic sulfate-reducing bacteria. *D. infernum* is a Gram-negative, non-motile sulfate reducer isolated from a North Sea petroleum reservoir, with optimal growth at 60 °C. It utilizes a range of organic acids and employs sulfite and thiosulfate as electron acceptors (Rees et al., 1995). *T. norvegica*, the closest known relative of *D. infernum*, was isolated from hot water associated with a North Sea oil field. It is Gram-negative, motile, thermophilic (optimal growth at 60 °C), and uses sulfate and sulfite as electron acceptors (Beeder et al., 1995). The presence of these taxa, both associated with hydrocarbon-rich petroleum systems, suggests that sulfate-reducing members of the *Syntrophobacterales* may contribute to hydrocarbon metabolism in this geothermal environment.

## 4 Conclusion

Biological methane oxidation was detected across Yellowstone hot spring systems spanning a wide range of pH and temperature conditions, revealing unexpectedly diverse—and in some cases extreme—physiological capabilities. The highest measured rate, 107 nmol C gdws^−1^·day^−1^, occurred in an acidic (pH 4.0), moderately hot (53 °C) system harboring bacterial aerobic methanotrophs of the phylum *Verrucomicrobia*, organisms known for thriving in acidic geothermal habitats.

In other systems, methane oxidation co-occurred with archaeal ammonia oxidizers, extending growing evidence that these taxa, like their bacterial counterparts, may be capable of methane oxidation and could represent an underappreciated methane sink in geothermal environments. Methane oxidation was also recorded at a high-temperature (89.9 °C), circumneutral (pH 7.3) site with detectable hydrocarbons but without canonical bacterial methanotrophs or archaeal ammonia oxidizers, implicating hydrocarbon-degrading organisms such as alkanotrophs as possible methane oxidizers in such extreme niches.

Strikingly, measurable oxidation rates were found even in systems where no known methane-oxidizing taxa were detected by our sequencing efforts. This discrepancy points toward the presence of uncharacterized or deeply divergent microbial lineages with the capacity for methane oxidation, possibly representing novel thermophilic metabolisms that are not captured by current genomic reference databases. Such lineages could fill unexplored functional roles in hydrothermal carbon cycling.

Together, these findings expand the recognized ecological and physiological range of methane-oxidizing microorganisms in continental hydrothermal systems, highlight previously overlooked methane sinks—including archaeal ammonia oxidizers and alkane degraders—and underscore the potential for discovering entirely new thermophilic methane oxidizers in extreme environments. Given the potency of methane as a greenhouse gas, identifying and characterizing these novel pathways is critical for refining our understanding of methane fluxes, both in geothermal systems and in global methane budgets.

## Data Availability

Shotgun metagenome sequencing data can be found in the National Center for Biotechnology Information Sequence Read Archive under the Bioproject, https://www.ncbi.nlm.nih.gov/bioproject/PRJNA1427733.

## Footnotes

1
https://worm-portal.asu.edu

2
https://www.ncbi.nlm.nih.gov/taxonomy

3
https://www.kbase.us

4
https://www.bioinformatics.babraham.ac.uk/projects/fastqc/
