# Supplementary material for: Pushing the upper temperature limit of methanotrophy in continental hydrothermal ecosystems, active biological methane oxidation in hot springs of Yellowstone National Park
Source: Front Microbiol. 2026 Mar 23;17:1736896. doi: 10.3389/fmicb.2026.1736896 (PMC13050887; doi:10.3389/fmicb.2026.1736896)
Supplement: Supplementary file 1 [file Data_Sheet_1.pdf]

Supplementary Material

Supplementary Table Captions

Supplementary Table 1. Field and laboratory measurements.

Supplementary Table 2. Log activity of aqueous chemical species.

Supplementary Table 3. Taxonomic classification of ASVs.

Supplementary Table 4. ASV sequence counts at each study site.

Supplementary Table 5. Relative abundance of 16S rRNA gene phylotypes grouped at the species level.

Supplementary Table 6. Summary of all metagenome assembled genomes, their completeness and their taxonomic classification.

Supplementary Table 7. Presence/absence and relative abundance of genes encoding putative monooxygenase in metagenome assembled genomes of Yellowstone hot springs reported in Colman et al. (2024).

Generative AI Statement

Generative AI was used to edit the manuscript text for clarity. ChatGPT version 5.0 (https://chatgpt.com) was used. The first and last prompts used were, "Please edit for clarity". AI edits were thoroughly examined for accuracy.
